# Changes in local brain function in mild cognitive impairment due to semantic dementia

**DOI:** 10.1111/cns.13621

**Published:** 2021-03-02

**Authors:** Liang Cui, Keliang Chen, Lin Huang, Jiawei Sun, Yating Lv, Xize Jia, Qihao Guo

**Affiliations:** ^1^ Department of Gerontology Shanghai Jiao Tong University Affiliated Sixth People’s Hospital Shanghai China; ^2^ Department of Neurology Huashan Hospital Fudan University Shanghai China; ^3^ School of Information and Electronics Technology Jiamusi University Jiamusi China; ^4^ Institute of Psychological Sciences Hangzhou Normal University Hangzhou China; ^5^ Zhejiang Key Laboratory for Research in Assessment of Cognitive Impairments Hangzhou China

**Keywords:** frequency‐dependent, mild cognitive impairment, resting‐state functional MRI, semantic dementia

## Abstract

**Aims:**

Mild cognitive impairment due to semantic dementia represents the preclinical stage, involving cognitive decline dominated by semantic impairment below the semantic dementia standard. Therefore, studying mild cognitive impairment due to semantic dementia may identify changes in patients before progression to dementia. However, whether changes in local functional activity occur in preclinical stages of semantic dementia remains unknown. Here, we explored local functional changes in patients with mild cognitive impairment due to semantic dementia using resting‐state functional MRI.

**Methods:**

We administered a battery of neuropsychological tests to twenty‐two patients with mild cognitive impairment due to semantic dementia (MCI‐SD group) and nineteen healthy controls (HC group). We performed structural MRI to compare gray matter volumes, and resting‐state functional MRI with multiple sub‐bands and indicators to evaluate functional activity.

**Results:**

Neuropsychological tests revealed a significant decline in semantic performance in the MCI‐SD group, but no decline in other cognitive domains. Resting‐state functional MRI revealed local functional changes in multiple brain regions in the MCI‐SD group, distributed in different sub‐bands and indicators. In the normal band, local functional changes were only in the gray matter atrophic area. In the other sub‐bands, more regions with local functional changes outside atrophic areas were found across various indicators. Among these, the degree centrality of the left precuneus in the MCI‐SD group was positively correlated with general semantic tasks (oral sound naming, word‐picture verification).

**Conclusion:**

Our study revealed local functional changes in mild cognitive impairment due to semantic dementia, some of which were located outside the atrophic gray matter. Driven by functional connectivity changes, the left precuneus might play a role in preclinical semantic dementia. The study proved the value of frequency‐dependent sub‐bands, especially the slow‐2 and slow‐3 sub‐bands.

## Graphical abstract

This study uses resting‐state fMRI with multiple sub‐bands and indicators to explore functional activity in patients with preclinical semantic dementia. The study find local functional changes in some regions distant from gray matter atrophy areas. Moreover, the left precuneus may play a role in the decline of semantic function.

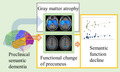

## 1 INTRODUCTION

Semantic dementia (SD) is a subtype of frontotemporal lobe degeneration,^1^ which is the leading cause of dementia in people under 65 years of age, especially in middle age.^2^ Because the typical damage is in the anterior temporal lobe, SD can also be regarded as a temporal variant of frontotemporal lobe degeneration. The main feature of SD is the loss of semantic memory domains,^1^, ^3^ but some patients initially present with personality and behavioral changes, such as apathy and disinhibition; the differences in SD symptoms are caused by lateralization.^4^ Typical SD symptoms often correspond to left anterior temporal lobe atrophy, although in some patients, atrophy begins on the right side.^5^, ^6^ This diversity of the initial symptoms makes it challenging to recruit and study patients with preclinical SD.

Local pathophysiological changes in neurodegenerative diseases exist before the emergence of severe clinical symptoms. Previous studies have found tau‐related pathological damage in the preclinical stage of frontotemporal lobe degeneration ^7^ and decreased local metabolism in mild cognitive impairment.^7^ Mild cognitive impairment is the preclinical stage of dementia; at this stage, cognitive decline is evident but it has not yet reached the criteria of dementia.^8^ Some patients diagnosed with mild cognitive impairment exhibit impaired semantic function, and during subsequent follow‐up are diagnosed with SD. These patients with mild cognitive impairment due to SD are suitable for the study of preclinical SD.

Neuroimaging plays a vital role in diagnosis. In the current diagnostic criteria, clinical symptoms and neuroimaging examination are both necessary, in which neuroimaging examination needs to have one of the following manifestations: (a) MRI dominant significant anterior temporal lobe atrophy; or (b) single‐photon emission computed tomography (SPECT) or PET dominant anterior temporal lobe hypoperfusion or hypometabolism.^3^ Therefore, MRI has always been an essential tool for studying SD.^9^ Structural MRI studies have revealed the extent of gray matter (GM) atrophy and its impact on the occurrence and development of disease.^10^, ^11^, ^12^ Moreover, functional connectivity studies using resting‐state functional magnetic resonance imaging (rs‐fMRI) have revealed the changes in brain network.^10^, ^13^ However, there are few studies on the changes in local brain activities. It is not clear whether there are changes in regional brain function in patients with SD, and whether the areas in which these changes exist are consistent with the known GM atrophy. Local functional indicators of rs‐fMRI can evaluate spontaneous neural activity from different aspects. The amplitude of low‐frequency fluctuation (ALFF), fractional amplitude of low‐frequency fluctuation (fALFF), percent amplitude of fluctuation (PerAF), and wavelet‐based amplitude of low‐frequency fluctuation (Wavelet‐ALFF)^14^, ^15^ can describe the amplitude of local spontaneous nerve activity. Regional homogeneity (ReHo) can describe the consistency of spontaneous neural activity in local brain regions and adjacent brain regions.^16^, ^17^ Degree centrality (DC) is used to describe the importance of local brain areas in global networks.^18^

Studies using rs‐fMRI have primarily focused on the fluctuation of blood oxygen level‐dependent (BOLD) signal in the low‐frequency band (0.01–0.08 Hz).^19^, ^20^, ^21^ However, such a relatively wide frequency band may not be sensitive enough due to noise or other reasons.^22^, ^23^ Therefore, researchers have divided the BOLD signal into four sub‐bands according to frequency: slow‐5 (0.01–0.027 Hz), slow‐4 (0.027–0.073 Hz), slow‐3 (0.073–0.198 Hz), and slow‐2 (0.198–0.25 Hz). These sub‐bands showed specific changes in different diseases.^24^, ^25^ In studies of some neurological or psychiatric diseases, the combination of different frequency bands has provided more refined results.^26^, ^27^, ^28^, ^29^

In this study, we selected patients who were diagnosed with semantic impaired mild cognitive impairment at the first visit and were diagnosed with SD in the subsequent follow‐up to obtain a preclinical SD study cohort. To increase the effectiveness of the study, we used different frequency bands combined with a variety of local functional indicators to reflect local brain function. Specifically, we conducted a voxel‐based morphometry (VBM) analysis and an exploratory rs‐fMRI analysis in patients with mild cognitive impairment due to SD. The rs‐fMRI sub‐bands included the main valuable frequency bands, that is, the normal band, slow‐2 band, slow‐3 band, slow‐4 band, and slow‐5 band. The fMRI indicators included ALFF, fALFF, PerAF, Wavelet‐ALFF, ReHo, and DC. The goals of this study were to explore the changes in local brain function in patients with mild cognitive impairment due to SD, to analyze the relationship between these changes and GM atrophy, and to evaluate the value of the combination of sub‐bands and multiple indicators. We hope that the results will help us to understand the characteristics of local brain functional changes in preclinical SD and aid in further longitudinal studies and subgroup analyses.

## 2 MATERIALS AND METHODS

### 2.1 Participants

#### 2.1.1 The mild cognitive impairment due to SD group (MCI‐SD group)

Twenty‐two patients diagnosed with mild cognitive impairment due to SD were included (eight men; age: mean = 61.59 years, standard deviation (s.d.) = 6.98 years, range = 46–70 years; number of years of full‐time education: mean = 11.9 years, s.d. = 3.35 years, range = 6–15 years; Mini‐Mental State Examination [MMSE]: mean = 25.2, s.d. = 1.47, range = 24–28). These patients showed mild cognitive impairment with impaired language ability at their first visit and were diagnosed with SD in the subsequent (at least one year later) follow‐up. All participants took part in the study at the outpatient department of Huashan Hospital and were recruited from 2013 to 2017. Participants underwent neuropsychological tests and brain MRI examinations. The inclusion criteria were as follows: (a) at the first visit, the Chinese version of the MMSE^30^ score ranged from 24 to 28, showing a decline in semantic function, with no obvious defect in professional ability, social ability, and living ability; (b) during subsequent follow‐up, the patient developed a worsening naming disorder and a word comprehension problem, with at least the following three characteristics: lack of object knowledge, surface dyslexia, less repetition ability, and less pronunciation ability, and hit the diagnostic criteria for SD^3^; and (c) manifestation of temporal lobe atrophy on MRI supporting the diagnosis of SD.^3^ Half of the cases completed the fluorodeoxyglucose (FDG)‐PET scan. The exclusion criteria were as follows: (a) patients diagnosed with non‐fluent/semantic variants of primary progressive aphasia (PPA) and logopenic PPA, or other types of dementia were excluded; (b) patients with a history of head injury, head surgery, neurological or mental diseases, or severe visual or hearing impairment; and (c) patients who could not undergo MRI due to metal implants or other reasons. 3D structural MRI was used for imaging diagnosis to exclude patients with severe brain diseases such as brain tumors, acute cerebral hemorrhage, cerebral ischemia, or non‐degenerative brain injury. 3D structure MRI was also visually inspected to evaluate the atrophy degree of the left and right temporal lobes by experienced physicians. In the MCI‐SD group, the number of patients with left atrophy and right atrophy was equal.

#### 2.1.2 The healthy control group (HC group)

Nineteen healthy participants were classified as the HC group (10 men; age: mean = 60.53 years, s.d. = 4.06 years, range = 51–69 years; the number of years of full‐time education: mean = 10.4 years, s.d. = 2.95 years, range = 2–15 years; MMSE: mean = 28.2, s.d. = 1.32, range = 27–30).

All participants were right‐handed, native Chinese‐speaking, with normal hearing and normal or corrected‐to‐normal vision. All participants signed written informed consent following the Declaration of Helsinki. The study was approved by the Institutional Review Committee of China Huashan Hospital (approval number 2009–195).

### 2.2 Behavioral data collection

We used the following six general semantic tasks: (a) Oral picture naming: participants were instructed to verbalize the name of an object whose picture was presented on a screen. (b) Oral sound naming (SN): a sound was played through headphones, and the participants were instructed to verbalize the name of the object whose sound was presented. (c) Picture associative matching: pictures of two objects were displayed at the bottom of a screen. Participants were instructed to judge which was semantically closer to the picture of an object at the top of the screen. (d) Word associative matching: Participants had to replace a picture with the appropriate Chinese name. (e) Word‐picture verification (WPV): participants were instructed to determine whether a name and a picture matched. (f) Naming to definition: After visual and audible presentation of an object, participants were instructed to verbalize its name.

To measure the cognitive ability of specific modal patterns, we conducted traditional neuropsychological tests, including the Boston Naming Test, Animal Fluency Test, Symbol digit modalities test, Shape trails test‐A, Shape trails test‐B, Rey Complex Figure Test copy, and Rey Complex Figure Test long delay recall.^31^, ^32^

### 2.3 Imaging data collection

All participants were scanned using a Siemens 3 T nuclear magnetic resonance scanner (Siemens) at Huashan Hospital in Shanghai to obtain structural 3D T1 MRI images and Resting‐State fMRI images. The 3D T1 image parameters were as follows: MPRAGE sequence, repetition time = 2300 ms, echo time = 2.98 ms, flip angle = 9°, matrix size = 240 × 256, field of view = 240 mm × 256 mm, slice number = 192 slices, slice thickness = 1 mm, voxel size = 1 × 1 × 1 mm.^3^ Resting‐State fMRI image parameters were as follows: echo‐planar imaging (EPI) sequence, transverse plane, repetition time = 2000 ms, echo time = 35 ms, flip angle = 90°, matrix size = 64 × 64, field of view = 256 mm × 256 mm, slice number = 33 slices, slice thickness = 4 mm, and voxel size = 4 mm × 4 mm × 4 mm. The scan obtained 200 slices and took a total of 400 s. During the entirety of the scan, the participants lay still in the scanner but remained awake with their eyes closed and were instructed not to think systematically.

### 2.4 Imaging data processing

#### 2.4.1 T1 imaging data

The 3D T1‐weighted images were processed using an optimized VBM protocol in Statistical Parametric Mapping 12 (SPM12) (http://www.fil.ion.ucl.ac.uk/spm/software/spm12/). Using the Montreal Neurological Institute (MNI) coordinates, segmentation and normalization were carried out to produce GM, white matter (WM), and cerebrospinal fluid (CSF) probability maps. GM maps were modulated by multiplying the intensity of each voxel by the local value derived from the deformation field and then smoothed using an 8 mm full width and half maximum (FWHM) kernel and used for further analysis.

#### 2.4.2 Resting‐State fMRI imaging data

The data were processed using SPM12 and RESTplus (http://restfmri.net/forum/restplus) toolkits.^33^ The first 10 time points were removed to allow the participant to adapt to the scanning noise and avoid the non‐equilibrium effects of magnetization. Slice timing adjustment and realignment for the correction of head motion were also conducted. The threshold of excessive head movement was defined as being when the head rotated more than 3° or shifted more than 3 mm in any direction during the entire scanning process. All participants were below this threshold and were included in the whole process. In the next step, through linear transformation, the individual structural images were coregistered to the mean functional image after head‐motion correction. The transformed structural images were segmented into GM, WM, and CSF using a unified segmentation algorithm. With the normalization parameters estimated in the process of unified segmentation, the functional volumes after head‐motion correction were spatially normalized to the MNI space and resampled to 3 mm isotropic voxels. Linear detrending processing was carried out to eliminate linear drift of the signal. Individual‐level regression analysis was used to minimize the effects of head movement (Friston24 model), WM signal noise, and CSF signal noise. For multi‐band analysis, we used a normal band (0.01–0.08 Hz) and the following four sub‐bands: slow‐5 (0.01–0.027 Hz), slow‐4 (0.027–0.073 Hz), slow‐3 (0.073–0.198 Hz), and slow‐2 (0.198–0.25 Hz).

#### 2.4.3 Computing ALFF, fALFF, Wavelet‐ALFF, and PerAF

The ALFF calculation used the fast Fourier transform to convert each time course to the frequency domain. Filtering was not required during pre‐processing. Subsequently, we calculated the average square root of the power spectrum at each frequency. The fALFF calculation was similar to the ALFF mentioned above. The fALFF was obtained by dividing the sum of the amplitudes in the target frequency band by the sum of the amplitudes in the whole frequency range. The Wavelet‐ALFF was calculated based on the continuous wavelet transform. The wavelet coefficients of all time points at each frequency point were summed to calculate the Wavelet‐ALFF, and the average coefficients on a given frequency band were obtained. The PerAF was calculated by measuring the percentage of BOLD fluctuation relative to the average BOLD signal strength at each time point and averaging the entire time series.

The ALFF, fALFF, PerAF, and Wavelet‐ALFF were standardized by dividing the value of each voxel by the global average. Finally, the standardized mALFF, mfALFF, mPerAF, and mWavelet‐ALFF graphs were spatially smoothed using a Gaussian kernel (FWHM = 4 mm). We calculated the indicators of the five frequency bands defined above.

#### 2.4.4 Computing ReHo and DC

We obtained the ReHo by calculating the Kendall coordination coefficient of the time process for each of the 27 nearest neighboring voxels. Standardization and spatial smoothing were carried out in the same way as the above indicators.

DC represents the sum of weights that showed node strength with a given voxel in the weighted graphs. For each voxel, the BOLD time course was extracted, and the Pearson correlation coefficients with every other voxel in the brain were calculated. A matrix of Pearson's correlation coefficients between a given voxel and all other voxels was obtained to construct the whole‐brain functional connectivity matrix for each voxel. An undirected adjacency matrix was then obtained by setting a threshold to each correlation at an r value more than 0.25. Then, the weighted DC was calculated as the sum of correlations exceeding this threshold. The DC value of each voxel was divided by the global mean of the DC values for standardization. Finally, the resulting matrices were smoothed with a Gaussian kernel (FWHM = 4 mm) to enable group comparisons.

### 2.5 Statistical analysis

We used SPSS (IBM SPSS Statistics, Version 26.0. IBM Corp) software for statistical analysis of demographic data and neuropsychological scale scores. Data were tested for normality using a Shapiro‐Wilk normality test. Age, education years, and MMSE scores conformed to a normal distribution, and a two‐sample *t*‐test was used to analyze the differences between groups; the chi‐square test was conducted for sex. The semantic battery (including Oral picture naming, Oral sound naming, Picture associative matching, Word associative matching, Word‐picture verification, Naming to definition, Boston naming test, Animal fluency test, Symbol digit modalities test, Shape trials test‐A, Shape trials test‐B, Complex figure test copy, Complex figure test recall) scores did not conform to the normal distribution, and these data were analyzed using nonparametric tests. The Mann‐Whitney U test was conducted to compare the difference in semantic task scores between the two groups. Spearman's rank correlation was used to estimate the correlation between fMRI indicators and semantic task scores.

To compare the volume of GM between the two groups, Data Processing and Analysis of Brain Imaging version 4.0 (DPABI, http://rfmri.org/DPABI) was used to carry out a two‐sample *t*‐test between the SD and HC groups. To examine the between‐group differences of ALFF, fALFF, PerAF, Wavelet‐ALFF, ReHo, and DC, two‐sample *t*‐tests were conducted between the SD and HC groups using DPABI v4.0. The mean relative displacements of the GM map and mean framewise displacement were used as covariates to reduce the influence of mixed variables in the statistical analysis.^34^ Each voxel's gray matter volume (GMV) was used as a covariate to reduce the influence of GM atrophy. Multiple comparison correction was performed based on the Gaussian random field theory (GRF; voxel‐wise, *p* < 0.001; cluster‐wise, *p* < 0.05, two‐tailed).

## 3 RESULTS

### 3.1 Neuropsychological performances

There were no statistical differences in age, sex, and education between the SD and HC groups.

The MMSE score of the MCI‐SD group was significantly lower than that of the HC group (25.2 ± 1.47 vs. 28.2 ± 1.32, *t*‐test, *p* < 0.001). In general semantic tasks, the oral picture naming (126.0 vs. 54.0, U test, *p* < 0.001), SN(26.0 vs. 8.0, U test, *p* < 0.001), picture associative matching (67.0 vs. 54.0, U test, *p* < 0.001), word associative matching (67.0 vs. 58.5, U test, *p* < 0.001), WPV (67.0 vs. 51.5, U test, *p* < 0.001), and naming to definition scores (59.0 vs. 22.5, U test, *p* < 0.001) of the MCI‐SD group were significantly lower than those of the HC group. In the traditional neuropsychological tests, the Boston Naming Test (22.0 vs. 7.0, U test, *p* < 0.001) and Animal Fluency Test (16.0 vs. 8.0, U test, *p* < 0.001) scores of the MCI‐SD group, which reflect semantic memory, were significantly decreased compared to those of the HC group. There was no significant difference between the two groups in the symbol digit modalities test and shape trails test‐A reflecting attention, shape trails test‐B reflecting executive function, Rey Complex Figure Test copy reflecting spatial ability, and Rey Complex Figure Test long delay recall reflecting non‐verbal episodic memory (*p* > 0.05) (Table 1).

**TABLE 1:** Demographic and neuropsychological performances

	HC group	MCI‐SD group	t/z‐values
Sex	8/19 (M)	10/22 (M)	χ^2^ = 0.829
Age (year)^a^	60.53 (4.06)	61.59 (6.98)	−0.584
Education years (year)^a^	10.37 (2.95)	11.91 (3.35)	−1.55
General cognition^a^			
MMSE (full score: 30)	28.2 (1.32)	25.2 (1.46)	−6.96^c^
Semantic battery (full score or unit)			
General semantic tasks^b^			
Oral picture naming (140)	126.00 (121.00, 131.00)	54.00 (39.25, 66.25)	−5.466^c^
Oral sound naming (36)	26.00 (25.00, 29.00)	8.00 (6.75, 12.25)	−5.383^c^
Picture associative matching (70)	67.00 (65.00, 68.00)	54.00 (50.00, 58.25)	−5.215^c^
Word associative matching (70)	67.00 (67.00, 68.00)	58.50 (49.00, 62.25)	−5.271^c^
Word‐picture verification (70)	67.00 (66.00, 68.00)	51.5 (44.75, 59.00)	−5.180^c^
Naming to definition (70)	59.00 (54.00, 63.00)	22.50 (11.50, 27.00)	−5.455^c^
Traditional neuropsychological tests^c^			
Boston naming test (30)	22.00 (19.00, 25.00)	7.00 (5.00, 9.00)	−5.450^c^
Animal fluency test (number)	16.00 (14.00, 18.00)	8.00 (5.75, 10.00)	−5.317^c^
Symbol digit modalities test (number)	39.00 (31.00, 47.00)	33.50 (21.75, 46.00)	−1.112
Shape trials test‐A (second)	53.00 (44.00, 51.00)	58.50 (51.00, 79.00)	−1.531
Shape trials test‐B (second)	144.00 (119.00, 176.00)	173.00 (130.75, 210.25)	−1.386
Complex figure test copy (36)	35.00 (33.00, 36.00)	34.50 (31.75, 36.00)	−0.698
Complex figure test recall (36)	16.00 (9.00, 22.00)	10.00 (5.00, 15.00)	−1.914

Note

The MCI‐SD group was compared with the HC group.

Abbreviations: HC group: healthy control; M, Male; MCI‐SD group: mild cognitive impairment due to semantic dementia group; MMSE, Mini‐Mental State Examination.

^a^ *t*‐test, data are represented as means (standard deviations).

^b^ Mann‐Whitney U test, data are presented as median (first quartile, third quartile).

^c^ *p* < 0.001 indicates statistical significance compared to the healthy controls.

### 3.2 Gray matter volume

**FIGURE 1:**
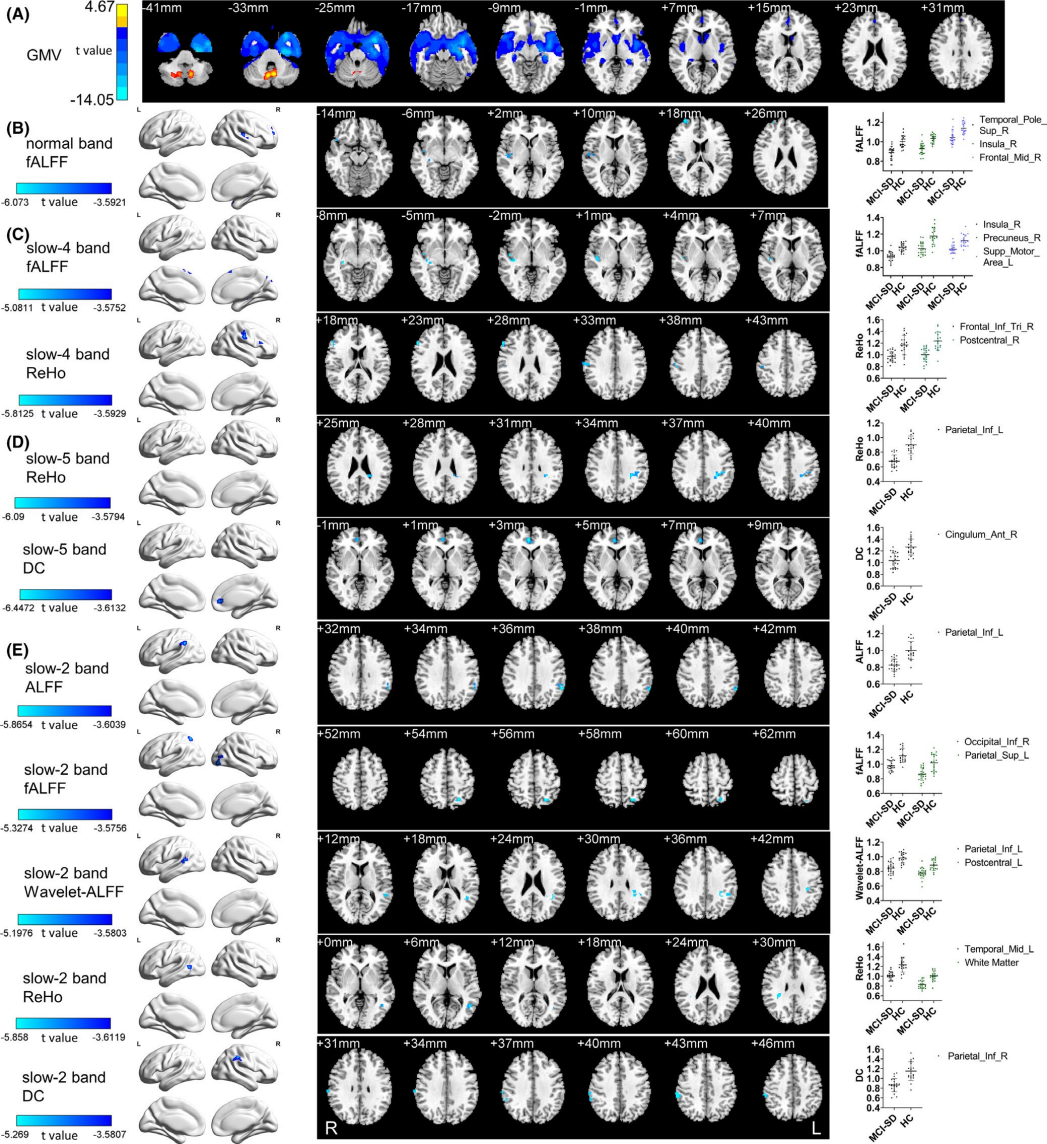
GMV differences and local functional changes in normal band, slow‐4, slow‐5, and slow‐2 band. The MCI‐SD group was compared with the HC group. (A) VBM analysis shows gray matter regions with GMV differences across groups. (B) In the normal band, the fALFF decreased in Temporal_Pole_Sup_R, Frontal_Inf_Orb_R, Temporal_Sup_R, Rolandic_Oper_R, Heschl_R, Insula_R, Frontal_Mid_R, Frontal_Sup_R. (C) In slow‐4 band, fALFF decreased in Temporal_Sup_R, Heschl_R, Cuneus_R, Precuneus_R, Parietal_Sup_R, Supp_Motor_Area. ReHo decreased in Frontal_Inf_Tri_R, Postcentral_R, SupraMarginal_R. (D) In slow‐5 band, ReHo decreased in Parietal_Inf_L. DC decreased in Cingulum_Ant_R, Frontal_Sup_Medial_R, Cingulum_Ant_L. (E) In slow‐2 band, ALFF decreased in Parietal_Inf_L, SupraMarginal_L. FALFF decreased in Occipital_Inf_R, Occipital_Mid_R, Temporal_Mid_R, Parietal_Sup_L. Wavelet‐ALFF decreased in Temporal_Mid_L, SupraMarginal_L, Temporal_Sup_L, Parietal_Inf_L, Angular_L, Postcentral_L, Parietal_Inf_L. ReHo decreased in Temporal_Mid_L, White Matter. DC decreased in SupraMarginal_R, Parietal_Inf_R. (GRF, voxel‐wise *p* < 0.001, cluster‐wise *p* < 0.05, two‐tailed). Brain region labels are taken from the Anatomical Automatic Labeling atlas (AAL). ALFF, amplitude of low‐frequency fluctuation; fALFF, fractional amplitude of low‐frequency fluctuation; Wavelet‐ALFF, wavelet‐based amplitude of low‐frequency fluctuation; ReHo, regional homogeneity; DC, degree centrality

Compared with the HC group, the MCI‐SD group showed decreased GMV in the bilateral inferior temporal gyrus, middle temporal gyrus, the pole of the superior temporal gyrus, the pole of the middle temporal gyrus, superior temporal gyrus, fusiform gyrus, insular lobe, parahippocampal gyrus, hippocampus, the orbital and triangular parts of the inferior frontal gyrus, medial superior frontal gyrus, caudate nucleus, basomedial amygdala, olfactory cortex, globus pallidus, anterior cingulate gyrus, rectus gyrus, and rolandic operculum; and increased GMV in the cerebellum (GRF corrected, cluster >465voxels, FWHMx 18.11 mm, FWHMy 20.13 mm, FWHMz 18.61 mm, voxel‐wise *p* < 0.001, cluster‐wise *p* < 0.05, two‐tailed. Figure 1).

### 3.3 Local functional changes in sub‐bands

We first carried out an analysis of the ordinary frequency normal band. We then analyzed the four sub‐bands according to frequency. Because the slow‐4 and slow‐5 bands coincide with the ordinary band to some extent, whereas the slow‐2 and slow‐3 bands are basically outside it, we divided the four sub‐bands into two groups.

#### 3.3.1 The normal band

In the normal band, decreased fALFF in the MCI‐SD group was observed in the right temporal pole, right superior temporal gyrus, and right dorsolateral superior frontal gyrus (extended to the middle frontal gyrus) (GRF corrected, cluster >27voxels, FWHMx 8.56 mm, FWHMy 8.75 mm, FWHMz, 8.35 mm, voxel‐wise *p* < 0.001, cluster‐wise *p* < 0.05, two‐tailed. Table 2, Figure 1). All the regions were located either in or adjacent to the areas of GM atrophy. There was no difference in ALFF, PerAF, Wavelet‐ALFF, DC, and ReHo between the two groups.

**TABLE 2:** The regions of difference between groups in the normal band, slow‐4 band, and slow‐5 band

	Norm band				Slow‐4				Slow‐5			
	Cluster (AAL)^a^	Peak (MNI)	Size	Peak intensity	Cluster (AAL)^a^	Peak (MNI)	Size	Peak intensity	Cluster (AAL)^a^	Peak (MNI)	Size	Peak intensity
fALFF	**cluster1**	30 18 −27	33	−6.073	**cluster1**	42 −21 −3	35	−4.8876	null			
	Temporal_Pole_Sup_R		19		Temporal_Sup_R		13					
	Frontal_Inf_Orb_R		8		Heschl_R		8					
	**cluster2**	42 −21 −3	60	−5.5679	Insula_R		1					
	Temporal_Sup_R		17		**cluster2**	9 −78 45	40	−5.0811				
	Rolandic_Oper_R		15		Cuneus_R		11					
	Heschl_R		15		Precuneus_R		11					
	Insula_R		6		Parietal_Sup_R		3					
	**cluster3**	27 54 30	29	−5.7277	**cluster3**	−3 15 66	34	−4.981				
	Frontal_Mid_R		17		Supp_Motor_Area_L		23					
	Frontal_Sup_R		9		Supp_Motor_Area_R		7					
ReHo					**cluster1**	57 27 27	30	−5.8125	**cluster1**	−30 −36 36	58	−6.09
					Frontal_Inf_Tri_R		29		Parietal_Inf_L		10	
					**cluster2**	63 −18 33	40	−4.7614				
					Postcentral_R		34					
					SupraMarginal_R		4					
DC					null				**cluster1**	6 42 3	27	−6.4472
									Cingulum_Ant_R		16	
									Frontal_Sup_Medial_R		8	
									Cingulum_Ant_L		3	

Note

All results were corrected for multiple comparisons (GRF, voxel‐wise *p* < 0.001, cluster‐wise *p* < 0.05, two‐tailed).

Abbreviations: AAL, anatomical automatic labeling atlas; DC, degree centrality; FALFF, fractional amplitude of low‐frequency fluctuation; MNI, Montreal Neurological Institute; ReHo, regional homogeneity.

^a^ The MCI‐SD group compared with the HC group. The cluster size was represented by the number of voxels.

#### 3.3.2 Slow‐4 and slow‐5 bands

In the MCI‐SD group, the brain regions with local functional changes began to spread in the slow‐4 and slow‐5 bands, and more indicators showed changes in these two sub‐bands. It is noteworthy that some indicators changed in regions distant from the areas of GM atrophy.

##### Slow‐4 band

fALFF was decreased in the right insula (extending to right superior temporal gyrus and right heschl gyrus), right precuneus (extending to right cuneus), and bilateral supplementary motor area in the MCI‐SD group compared to that in the HC group(GRF corrected, cluster >24voxels, FWHMx 8.18 mm, FWHMy 8.36 mm, FWHMz 8.05 mm, voxel‐wise *p* < 0.001, cluster‐wise *p* < 0.05, two‐tailed. Table 2. Figure 1). ReHo was decreased in the right triangular inferior frontal gyrus, the right postcentral gyrus, and the right superior marginal gyrus in the MCI‐SD group compared to that of the HC group (GRF corrected, cluster >29voxels, FWHMx 8.72 mm, FWHMy 9.19 mm, FWHMz 8.73 mm, voxel‐wise *p* < 0.001, cluster‐wise *p* < 0.05, two‐tailed. Table 2. Figure 1).

##### Slow‐5 band

In the MCI‐SD group, ReHo was decreased in the left superior parietal gyrus compared to that in the HC group(GRF corrected, cluster >29voxels, FWHMx 8.72 mm, FWHMy 9.09 mm, FWHMz 8.59 mm, voxel‐wise *p* < 0.001, cluster‐wise *p* < 0.05, two‐tailed. Table 2. Figure 1). DC was decreased in the right anterior cingulate in the MCI‐SD group compared to that in the HC group (GRF corrected, cluster >25voxels, FWHMx 8.21 mm, FWHMy 8.44 mm, FWHMz 8.18 mm, voxel‐wise *p* < 0.001, cluster‐wise *p* < 0.05, two‐tailed. Table 2. Figure 1).

#### 3.3.3 Slow‐2 and slow‐3 bands

In the slow‐2 and slow‐3 bands, the MCI‐SD group showed more brain regions with local functional changes on a further increased number of indicators. In these two sub‐bands, more brain regions distant from the atrophic areas showed local functional changes, and these were primarily distributed in the parietal and occipital lobes. It is noteworthy that in the slow‐3 band, all the local functional indicators used in this study showed changes in multiple brain regions. Moreover, in the slow‐3 band, the DC of the left precuneus, which is distant from the atrophic areas, showed a correlation with semantic function.

##### Slow‐2 band

In the slow‐2 band, there were differences in some brain regions between the two groups in ALFF, fALFF, Wavelet‐ALFF, ReHo, and DC. In the MCI‐SD group, ALFF was decreased in the left inferior parietal angular gyrus (GRF corrected, cluster >23voxels, FWHMx 7.92 mm, FWHMy 8.45 mm, FWHMz 7.67 mm, voxel‐wise *p* < 0.001, cluster‐wise *p* < 0.05, two‐tailed. Table 3. Figure 1). fALFF was decreased in the right inferior occipital gyrus (extending to the right middle occipital gyrus) and left superior parietal gyrus (GRF corrected, cluster >25voxels, FWHMx 8.35 mm, FWHMy 8.51 mm, FWHMz 8.06 mm, voxel‐wise *p* < 0.001, cluster‐wise *p* < 0.05, two‐tailed. Table 3. Figure 1). Wavelet‐ALFF was decreased in the left inferior parietal lobule (extending to the left middle temporal gyrus) and left postcentral gyrus (GRF corrected, cluster >23voxels, FWHMx 7.92 mm, FWHMy 8.52 mm, FWHMz 7.75 mm, voxel‐wise *p* < 0.001, cluster‐wise *p* < 0.05, two‐tailed. Table 3. Figure 1). ReHo was decreased in the left middle temporal gyrus and left WM of the inferior parietal lobule (GRF corrected, cluster >23voxels, FWHMx 7.95 mm, FWHMy 8.34 mm, FWHMz 7.81 mm, voxel‐wise *p* < 0.001, cluster‐wise *p* < 0.05, two‐tailed, Table 3. Figure 1). DC was decreased in the right inferior parietal lobule(extending to the right supramarginal gyrus) (GRF corrected, cluster >23voxels, FWHMx 8.25 mm, FWHMy 8.33 mm, FWHMz 7.58 mm, voxel‐wise *p* < 0.001, cluster‐wise *p* < 0.05, two‐tailed. Table 3. Figure 1). There was no difference in PerAF between the two groups.

**TABLE 3:** The regions of difference between the two groups in the slow‐2 and slow‐3 bands

	Slow‐2 band				Slow‐3 band			
	Cluster (AAL)^a^	Peak (MNI)	Size	Peak intensity	Cluster (AAL)^a^	Peak (MNI)	Size	Peak intensity
ALFF	**cluster1**	−60 −51 39	25	−5.8654	**cluster1**	−42 −51 21	60	−5.3433
	Parietal_Inf_L		14		Temporal_Mid_L		12	
	SupraMarginal_L		6		SupraMarginal_L		12	
					Angular_L		4	
					Parietal_Inf_L		4	
					**cluster2**	−33 −45 33	37	−5.3631
					Postcentral L		4	
fALFF	**cluster1**	45 −87 −6	32	−4.6702	**cluster1**	−12 36 0	68	−5.4545
	Occipital_Inf_R		15		Insula_L		10	
	Occipital_Mid_R		11		Caudate_L		6	
	Temporal_Mid_R		3		Putamen_L		5	
	**cluster2**	−27 −63 57	25	−5.3274				
	Parietal_Sup_L		25					
PerAF	null				**cluster1**	57 6 21	75	−5.2406
					Frontal_Inf_Tri_R		31	
					Frontal_Inf_Oper_R		28	
					Precentral_R		9	
					**cluster2**	30 36 18	39	−4.8035
					Frontal_Mid_R		4	
					Cingulum_Ant_R		4	
Wavelet‐ALFF	**cluster1**	−42 −45 24	60	−4.6977	**cluster1**	−42 −51 21	47	−5.058
	Temporal_Mid_L		19		Temporal_Mid_L		11	
	SupraMarginal_L		10		SupraMarginal_L		10	
	Temporal_Sup_L		8		Parietal_Inf_L		5	
	Parietal_Inf_L		5		Angular_L		4	
	Angular_L		4		**cluster2**	−30 −45 30	32	−5.5499
	**cluster2**	−30 −45 30	42	−5.1976	Postcentral_L		4	
	Postcentral_L		5					
	Parietal Inf L		3					
ReHo	**cluster1**	−48 −60 6	25	−5.1417	**cluster1**	69 −21 −27	36	4.8644
	Temporal_Mid_L		24		Temporal_Inf_R		20	
	**cluster2**	33 −36 30	28	−5.858	Temporal_Mid_R		8	
	White Matter				Fusiform_R		3	
	Parietal lobe		19					
	Frontal lobe		9					
	Inferior Parietal Lobule		6					
DC	**cluster1**	60 −36 45	43	−5.269	**cluster1**	6 −51 39	146	−5.5421
	SupraMarginal_R		37		Precuneus_L		71	
	Parietal_Inf_R		3		Precuneus_R		69	
					Cingulum_Mid_L		3	
					**cluster2**	−9 −57 54	42	−5.1327
					Precuneus_L		38	
					Precuneus_R		4	

Note

All results were corrected for multiple comparisons (GRF, voxel‐wise *p* < 0.001, cluster‐wise *p* < 0.05, two‐tailed).

Abbreviations: AAL, anatomical automatic labeling atlas; ALFF, amplitude of low‐frequency fluctuation; DC, degree centrality; fALFF, fractional amplitude of low‐frequency fluctuation; MNI, Montreal Neurological Institute; PerAF, percent amplitude of fluctuation; ReHo, regional homogeneity; Wavelet‐ALFF, wavelet‐based amplitude of low‐frequency fluctuation.

^a^ The MCI‐SD group compared with the HC group. The cluster size was represented by the number of voxels.

##### Slow‐3 band

**FIGURE 2:**
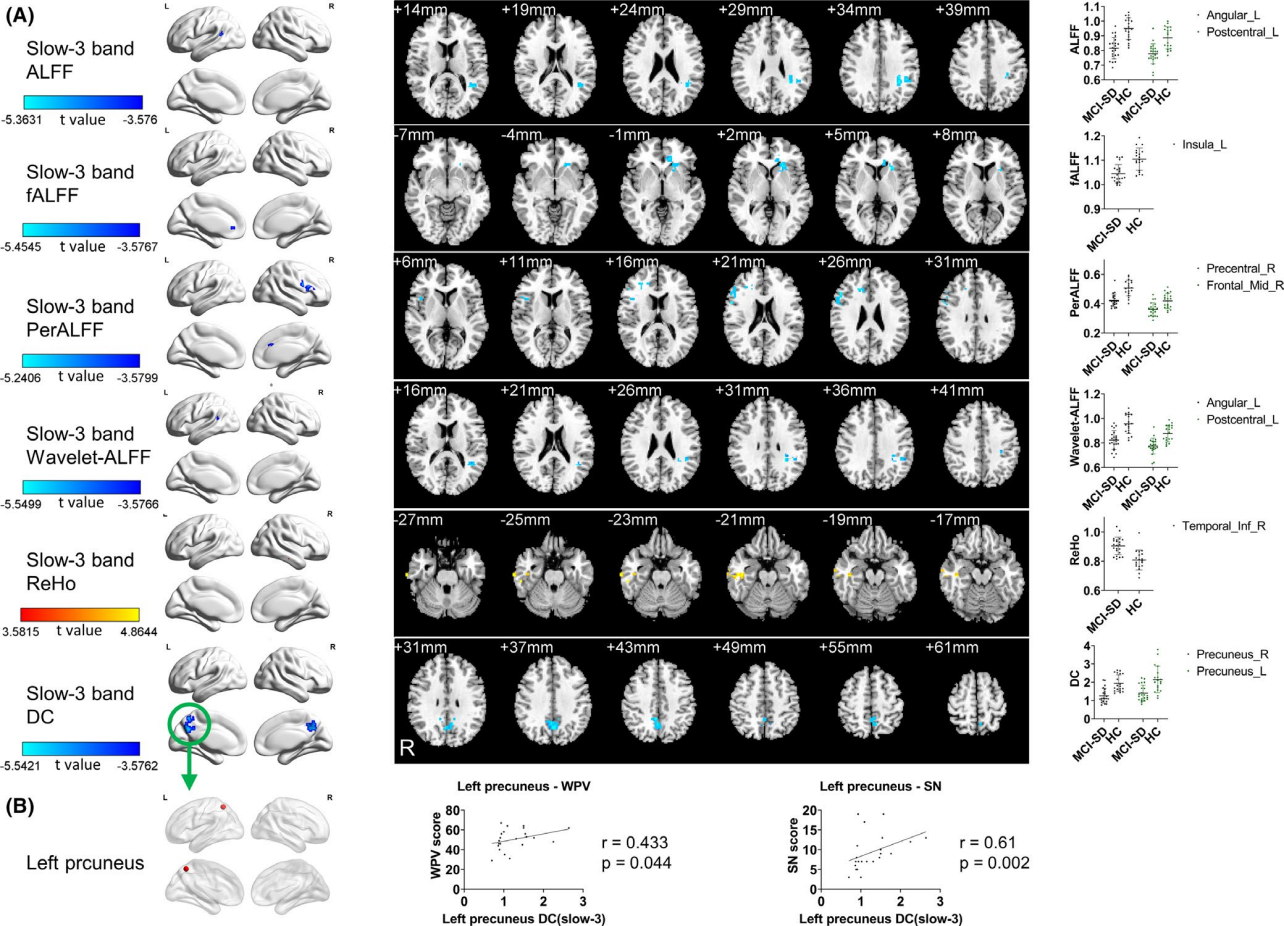
Local functional changes in slow‐3 band and correlation analysis. (A) The MCI‐SD group compared with the HC group in the slow‐3 band. ALFF decreased in Temporal_Mid_L, SupraMarginal_L, Angular_L, Parietal_Inf_L, Postcentral_L. FALFF decreased Insula_L, Caudate_L, Putamen_L. PerAF decreased in Frontal_Inf_Tri_R, Frontal_Inf_Oper_R, Precentral_R, Frontal_Mid_R, Cingulum_Ant_R. Wavelet‐ALFF decreased in Temporal_Mid_L, SupraMarginal_L, Parietal_Inf_L, Angular_L, and Postcentral_L. ReHo increased in the Temporal_Inf_R, Temporal_Mid_R, and Fusiform_R. DC decreased in the Precuneus_L, Precuneus_R, and Cingulum_Mid_L (GRF, voxel‐wise *p* < 0.001, cluster‐wise *p* < 0.05, two‐tailed). (B) Correlation analysis of DC in the left precuneus with WPV and SN. WPV, word‐picture verification, SN, oral sound naming. The dots in the dot graph were the values of the peak points of clusters. Brain region labels are taken from the Anatomical Automatic Labeling atlas (AAL). ALFF, amplitude of low‐frequency fluctuation; fALFF, fractional amplitude of low‐frequency fluctuation; PerAF, percent amplitude of fluctuation; Wavelet‐ALFF, wavelet‐based amplitude of low‐frequency fluctuation; ReHo, regional homogeneity; DC, degree centrality

In the slow‐3 band, there were differences in some brain regions between the two groups in ALFF, fALFF, PerAF, Wavelet‐ALFF, ReHo, and DC. In the MCI‐SD group, ALFF was decreased in the left angular gyrus (extending to left middle temporal gyrus and left superior marginal gyrus) and left inferior parietal gyrus (GRF corrected, cluster >23voxels, FWHMx 7.89 mm, FWHMy 8.49 mm, FWHMz 7.79 mm, voxel‐wise *p* < 0.001, cluster‐wise *p* < 0.05, two‐tailed. Table 3. Figure 2). fALFF was decreased in the left insular(GRF corrected, cluster >37voxels, FWHMx 9.98 mm, FWHMy 10.16 mm, FWHMz 9.66 mm, voxel‐wise *p* < 0.001, cluster‐wise *p* < 0.05, two‐tailed. Table 3. Figure 2). PerAF was decreased in the right precentral gyrus (extending to the right triangular part of the inferior frontal gyrus and right opercular part of the inferior frontal gyrus) and the middle frontal gyrus (GRF corrected, cluster >37voxels, FWHMx 9.76 mm, FWHMy 10.49 mm, FWHMz 9.41 mm, voxel‐wise *p* < 0.001, cluster‐wise *p* < 0.05, two‐tailed. Table 3. Figure 2). Wavelet‐ALFF was decreased in the left angular (extending to the left middle temporal gyrus and left superior marginal gyrus) and left postcentral gyrus (GRF corrected, cluster >23voxels, FWHMx 7.89 mm, FWHMy 8.49 mm, FWHMz 7.79 mm, voxel‐wise *p* < 0.001, cluster‐wise *p* < 0.05, two‐tailed. Table 3, Figure 2). ReHo was increased in the right inferior temporal gyrus (GRF corrected, FWHMx 8.38 mm, cluster >27voxels, FWHMy 9.00 mm, FWHMz_8.56 mm, voxel‐wise *p* < 0.001, cluster‐wise *p* < 0.05, two‐tailed. Table 3, Figure 2). DC decreased in the bilateral precuneus (GRF corrected, cluster >23voxels, FWHMx 8.13 mm, FWHMy 8.32 mm, FWHMz 7.72 mm, voxel‐wise *p* < 0.001, cluster‐wise *p* < 0.05, two‐tailed. Table 3, Figure 2).

To further explore the significance of local functional changes in these regions, we chose the left precuneus, which far from the atrophic area, as the region of interest (ROI). The Cluster2 (peak coordinate: −9, −57, 54) in the combination of slow‐3 band and DC represents the left precuneus. We defined the ROI as a sphere with the peak coordinate as the origin and radius of 6 mm. The Spearman rank correlation analysis with the general semantic tasks showed that the DC of the left precuneus was positively correlated with WPV (r = 0.433, *p* = 0.044) and SN (r = 0.61, *p* = 0.002) (Figure 2).

The unedited original pictures of the result corrected by multiple comparisons output by DPABI (Supplementary File 1) and the T‐Maps (Supplementary File 2) are provided in the Supplementary File.

## 4 DISCUSSION

We reviewed the mild cognitive impairment stage of patients diagnosed with SD to establish a study cohort of mild cognitive impairment due to SD and study preclinical SD. Analysis of the patients’ neuropsychological scales showed that they had cognitive decline limited to semantic function. We used VBM analysis to obtain the areas of GM atrophy and used GMV as a covariate in the subsequent rs‐fMRI analysis to eliminate the effect of GM atrophy on local brain function. In the rs‐fMRI analysis, we used an exploratory approach that combined frequency‐dependent sub‐bands with multiple indicators. Our study showed that there were more local functional changes in the sub‐bands than in the normal band, especially in the slow‐2 and slow‐3 bands. Some brain regions with local functional changes identified in the sub‐bands were distant from the areas of GM atrophy. Among these regions, the left precuneus had a close relationship with semantic functions, and we think this relationship was likely driven by changes in network connectivity.

### 4.1 Neuropsychological performance

The comparison of the neuropsychological tests showed that the performance of the MCI‐SD group in the general semantic tasks and semantic memory tasks was worse than that of the HC group. However, there was no significant difference in attention, executive function, spatial ability, and non‐verbal episodic memory between the two groups. These results showed that the decline of cognitive function in patients with mild cognitive impairment due to SD was primarily caused by semantic impairment, while the function in the other cognitive domains was roughly retained.

### 4.2 Functional changes in the atrophic areas

We identified local functional changes in multiple regions. Some of these regions, mostly located in the temporal lobe, frontal lobe, and cingulate gyrus, were in or adjacent to the areas of GM atrophy. The temporal pole is regarded as a critical area of abnormal connection in SD.^35^, ^36^ The orbitofrontal gyrus, which is close to the temporal pole and receives its signal input, is also a typical area of GM atrophy and reduced metabolism in SD.^4^, ^37^ Previous studies have confirmed that disconnection of the frontal lobe in the semantic network is one of the vital connectivity changes in SD.^38^

There are few studies on the cingulate gyrus in SD to this end. Cingulate gyrus dysfunction exists in a wide range of neurological and mental disorders. Some diffusion tensor imaging studies have reported that the structure of the cingulate gyrus in patients with frontotemporal dementia is damaged.^39^, ^40^ In a previous fMRI study, the connectivities of the limbic system, including the cingulate gyrus, were significantly decreased in patients with frontotemporal dementia.^41^ In frontotemporal dementia, there is a significant weakening of the salient network, which connects the frontal lobe and limbic system, and is characterized by communication between the anterior cingulate gyrus, insular, striatum, and amygdala.^42^ These studies primarily focused on the behavioral variant frontotemporal lobe degeneration subtype. However, in a recent study of the composition of the whole‐brain language network,^43^ the cingulate gyrus (including anterior and posterior) was found to play an important role.

It is generally believed that GM atrophy in SD is more significant on the left side^11^, ^12^; however, in our study, the local functional changes in the atrophic area were more significant on the right side. After visually inspected the temporal lobe atrophy of structural MRI, in the MCI‐SD group, the number of patients with left atrophy and right atrophy was equal. Unlike the predominance of left SD in most SD cohorts in the past, our preclinical SD cohort did not have obvious laterality. Therefore, local functional changes were found on both the left and right sides. Considering that GMV was used as a covariate in the rs‐fMRI statistical process, we think this may indicate that after excluding the laterality of GM atrophy, the local functional changes on the right side are more obvious than those on the left side in the GM atrophy area.

### 4.3 Functional changes distant from the atrophic areas

Local functional changes also occurred in some regions distant from the GM atrophic areas, indicating that they were not caused by GM atrophy. These regions were found in different sub‐bands with various indicators.

In the slow‐4 and slow‐5 bands, there were local functional changes in the right cuneus, right precuneus, and bilateral supplementary motor area (fALFF decreased in the slow‐4 band), and the left inferior parietal lobe (ReHo decreased in the slow‐5 band). As a region of visual cortex,^44^ the cuneus is also a part of a large‐scale network. This network integrates articulatory, auditory, and visual areas; its role is to produce, listen, and read lists of words.^45^ In previous studies, supplementary motor areas were considered to be related to motor language^46^, ^47^ and were involved in orthographic, phonological, and lexical‐semantic tasks.^48^ The role of supplementary motor areas in language function may be achieved through the connection with the superior temporal gyrus.^7^ The parietal lobe is an important part of the temporal/parietal/occipital module, which is an integral part of the network topology changes in SD and is related to semantic functional impairment.^49^ A meta‐analysis also showed that the left inferior parietal lobe engages in social cognition and language function, and the theory of mind and language‐related processing facets are inextricably linked to this region.^50^

In the slow‐2 and slow‐3 bands, the local functional changes in non‐atrophic areas were further extended to the left superior parietal gyrus (ALFF decreased in the slow‐2 band), right inferior occipital gyrus (fALFF decreased in the slow‐2 band), and precuneus (DC decreased in the slow‐3 band).

It was previously considered that the visual word form area is located in the occipitotemporal sulcus.^51^ However, an fMRI study recently found that the inferior and middle occipital gyri responded more strongly to Chinese characters than to visual images; this region plays a critical role in processing and representing the category information for words.^52^ Our study showed that the change was in the right inferior and middle occipital gyrus, which differs from the result of their study, which showed it was bilateral but mainly on the left side. The reason for this difference should be studied further in a subgroup study of left and right SD to determine whether it is related to the laterality of SD. There have been few studies regarding the superior parietal gyrus and semantic function. An fMRI study showed that this region is involved in the sublexical conversion of orthographic input into phonological codes in naming Chinese.^53^ Thus, the role of this region in SD should be explored further.

In the MCI‐SD group, the ReHo of the slow‐3 band increased in the right temporal lobe. The neuropathological hub in SD patients is on the left temporal lobe. Therefore, in the temporal lobe, as a critical area of semantic function, may appear functional compensation on the right side. ReHo reflects the consistency of local neural activity and does not depend on the number of remaining neurons and the activity intensity. Therefore, the increase of ReHo in the right temporal lobe may be a compensatory manifestation.

### 4.4 The left precuneus and semantic function

Among all the regions in which local functional changes were observed distant from the atrophic areas, the most important finding was the left precuneus. The precuneus is one of the main centers of the brain. It is an important node of the default mode network, but there are few studies on its relationship with SD, which may be caused by its location is hidden and far away from the typical SD gray matter atrophy areas. Our study found that the precuneus showed decreased fALFF in the slow‐4 band and decreased DC in the slow‐3 band, suggesting that it was affected in mild cognitive impairment due to SD. The correlation analysis found that the DC of the left precuneus was positively correlated with WPV and SN, and the correlation with SN survived after Bonferroni correction.

Some studies have suggested that the precuneus is involved in extended face recognition^54^, ^55^ and participates in facial emotion perception and context integration.^56^ Based on a new neural network analysis model,^57^ researchers found that the abnormality of precuneus combines with some risk genes has a good recognition ability for MCI and AD.^58^, ^59^ A previous study showed that a decrease in confrontation naming task scores in patients with early frontotemporal lobe degeneration was associated with decreased fMRI signals in the precuneus.^60^ The study reported that the effect was similar to that observed in patients with Alzheimer's disease and mild cognitive impairment, but the difference was not related to GM atrophy in the precuneus in patients with early frontotemporal lobe degeneration. Therefore, they hypothesized that default mode network inactivation was not related to local atrophic pathological changes, but to decreased connectivity. DC is a local indicator of connectivity, so our results verified this hypothesis. SD is a suitable model for semantic research, and the relationship between the precuneus and semantic function is worthy of further study in patients with SD. Furthermore, our results suggest to focus on DC in the slow‐3 band.

### 4.5 Value of the combination of sub‐bands and multiple indicators

We used the slow‐2, slow‐3, slow‐4, and slow‐5 sub‐bands in addition to the ordinary frequency band (ie, the normal band). The frequency range of each sub‐band is narrower than that of the normal band, while the total range is far greater. In the normal band, only limited regions with decreased fALFF values were observed in the atrophic areas; however, when the combination of sub‐bands and multiple indicators was added to the analysis, more local functional changes were observed (Table 4).

**TABLE 4:** Summary of sub‐bands and indicators combination

	ALFF	fALFF	PerAF	Wavelet‐ALFF	ReHo	DC
Normal band						
Sensitivity		√				
Inside/outside the atrophic area		Inside				
Slow‐5						
sensitivity					√	√
Inside/outside the atrophic area					Outside	Inside
Slow‐4						
sensitivity		√			√	
Inside/outside the atrophic area		Inside & outside			Inside & outside	
Slow‐3						
sensitivity	√	√	√	√	√	√
Inside/outside the atrophic area	Inside & outside	Inside	Inside	Inside & outside	Inside	Outside
Slow‐2						
sensitivity	√	√		√	√	√
Inside/outside the atrophic area	Outside	Outside		Inside & outside	Inside & outside	Outside

Note

√: Functional changes were found in this combination.

Abbreviations: ALFF, amplitude of low‐frequency fluctuation; DC, degree centrality; fALFF, fractional amplitude of low‐frequency fluctuation; PerAF, percent amplitude of fluctuation; ReHo, regional homogeneity; Wavelet‐ALFF, wavelet‐based amplitude of low‐frequency fluctuation.

The slow‐4 and slow‐5 bands approximately divide the ordinary frequency band into two. In these two bands, indicators changed in regions distant from the atrophic areas, including a decrease in fALFF in the right cuneus, right precuneus, right supplementary, and motor area; decreased ReHo in the left inferior parietal lobe; and decreased DC in the anterior cingulum. ReHo reflects the uniformity of local neural activity, whereas DC is a local indicator that can reflect the properties of the network. These two indicators added new dimensions to the evaluation of functional activity, allowing us to present our findings more comprehensively and holistically.

The frequencies of the slow‐2 and slow‐3 bands are higher than that of the ordinary band, but can still be obtained from BOLD signals. If we had abandoned these frequency ranges in the study, we might have missed some valuable information. From our results, these two bands did identify changed regions in most indicators.

It is noteworthy that wavelet‐ALFF showed high consistency in the slow‐2 band and slow‐3 bands, especially in the middle temporal gyrus, supramarginal gyrus, and inferior parietal angular gyrus, since these regions form Wernicke's area. The exact role of Wernicke's area, including the role of the supramarginal gyrus in aphasia, is still controversial. Some studies have shown that it is closely related to aphasia and comprehension disorder^61^, ^62^; however, it has also been proposed that it only plays the role of phonetic representation rather than language comprehension.^63^ If future studies use rs‐fMRI in SD queues to explore this area further, our results suggest that focusing on Wavelet‐ALFF in slow‐2 and slow‐3 bands may be useful. Wavelet‐ALFF is different from other fMRI indicators in that it is obtained by wavelet transform instead of the fast Fourier transform. Wavelet‐ALFF has strong adaptability to the local characteristics or instability of data and has better sensitivity and repeatability in the higher frequency band than in conventional frequency bands.^15^

In the slow‐3 band, PerAF decreased in the frontal lobe and limbic system. PerAF is an indicator that reflects the percentage fluctuation of rs‐fMRI and has good repeatability.^14^ According to our results, PerAF may be more suitable for analysis in slow‐3.

Both the slow‐2 band and the slow‐3 band showed ReHo changes in the temporal lobe. ReHo decreased in the right middle temporal gyrus in the slow‐2 band but increased in the left superior temporal gyrus and middle temporal gyrus in the slow‐3 band. The results showed lateralization in the uniformity of neural activity in the temporal lobe. ReHo in some brain regions of the WM showed decreases in the slow‐2 band. This result was consistent with the fact that slow‐2 is more sensitive to WM than other frequency bands.^24^

In summary, we think that the combination of sub‐bands and multiple indicators is valuable. At present, diseases with brain atrophy or other structural changes, including frontotemporal lobe degeneration,^64^, ^65^ AD,^66^ Parkinson's disease,^67^ and cerebral microvascular disease,^68^ mainly rely on structural MRI for diagnosis and differentiation. For the early stages of these diseases, such as the MCI stage, studies using RS‐fMRI mainly focused on changes in functional connectivity,^69^, ^70^, ^71^ and there are few studies on changes in local functional activities. We speculate that this may be due to the relatively slight pathological changes in the early stage of the disease, which leads to the insensitivity of the local functional indicators to reflect the nerve damage. A recent study on the interaction between gut microbiota with the local brain activity of amnestic MCI patients found different interaction types in different sub‐bands.^72^ This study supports our results. We believe that the sub‐bands combined with multiple indicators can make rs‐fMRI more sensitive and can reflect more changes. It may be worth promoting in the study of local functional activities in the early stages of brain diseases.

## 5 CONCLUSION

Patients with mild cognitive impairment due to SD showed mild cognitive decline caused by semantic impairment and were considered to be at the preclinical stage of SD. At this stage, GM atrophy is present, mainly in the temporal and frontal lobes. Local functional changes are evident at the same time, some of which are located in regions distant from the atrophic areas, suggesting that the changes in local function in mild cognitive impairment due to SD do not depend on GM atrophy. In non‐atrophic areas, the left precuneus may play a critical role in the decline of semantic function. Finally, the combination of sub‐bands and multiple indicators is valuable in rs‐fMRI.

## 6 LIMITATIONS

This study had some limitations. Left and right SD subgroup analyses could not be performed because the sample size was small. Therefore, although we found that some left and right symmetrical brain regions showed distinct differences in different frequency bands, the reasons and clinical significance of these differences were not studied further. If the sample size can be expanded, relevant research in future studies can be considered.

## COMPETING INTERESTS

The authors report no competing interests.

## Supporting information

Supplementary Material

## Data Availability

Data available on request from the authors.
