# Supplementary material for: Changes in local brain function in mild cognitive impairment due to semantic dementia
Source: CNS Neurosci Ther. 2021 Mar 2;27(5):587–602. doi: 10.1111/cns.13621 (PMC8025655; doi:10.1111/cns.13621)
Supplement: Supplementary file 1 — Supplementary Material [file CNS-27-587-s001.pdf]

Supplementary file 1. The unedited original picture of the result corrected by multiple comparisons output by DPABI

normal band Falff, full unedited image for Figure 1.

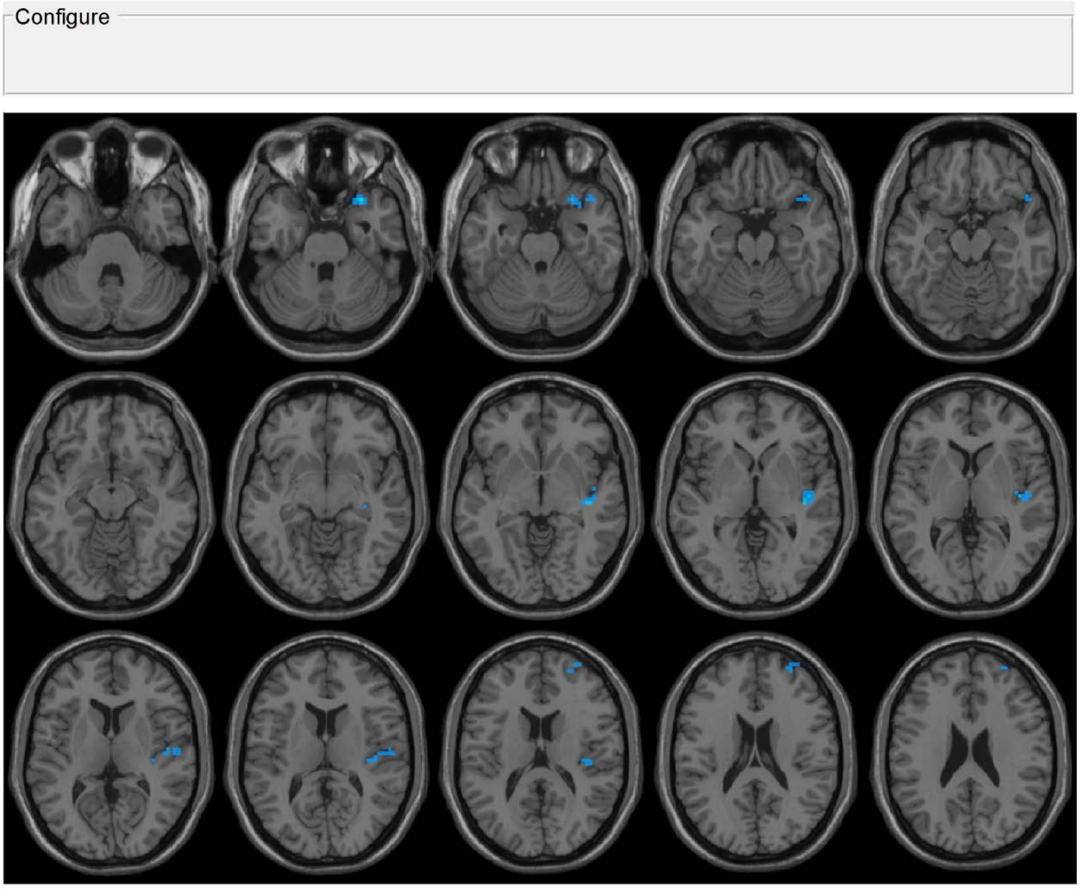

slow4 fALFF, full unedited image for Figure 1.

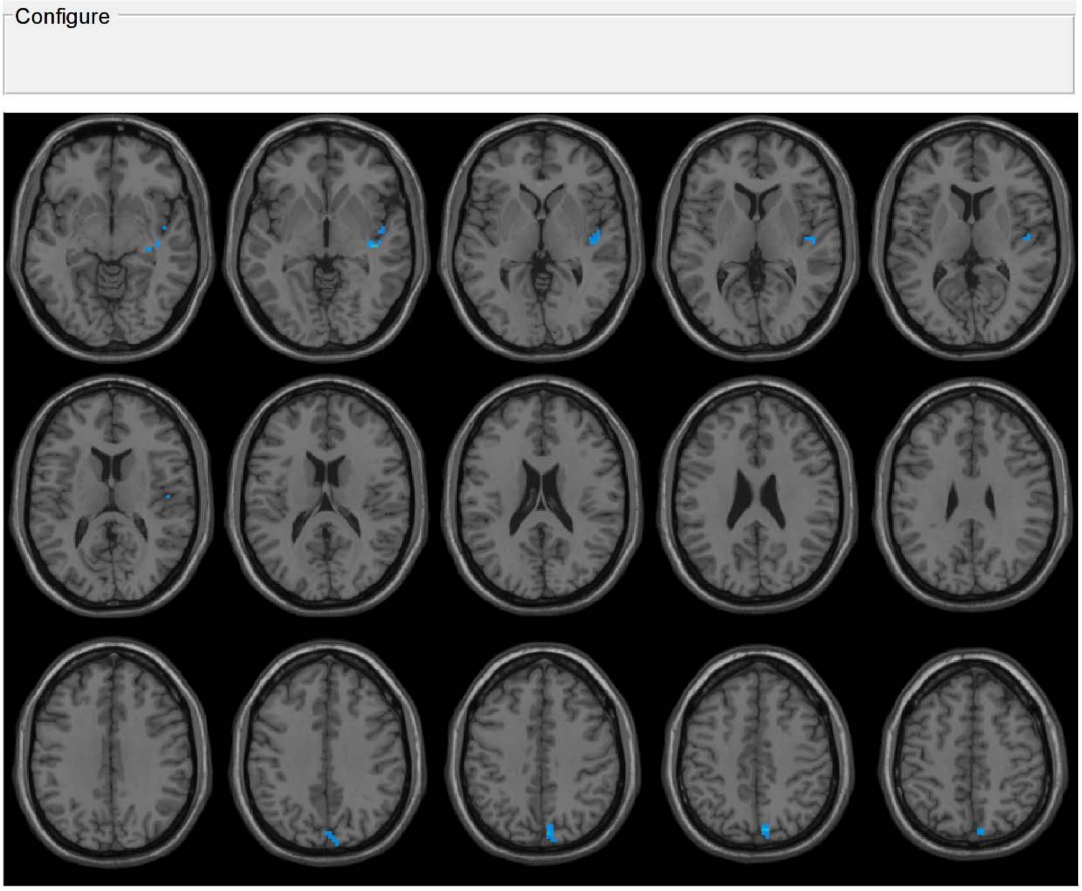

slow4 ReHo, full unedited image for Figure 1.

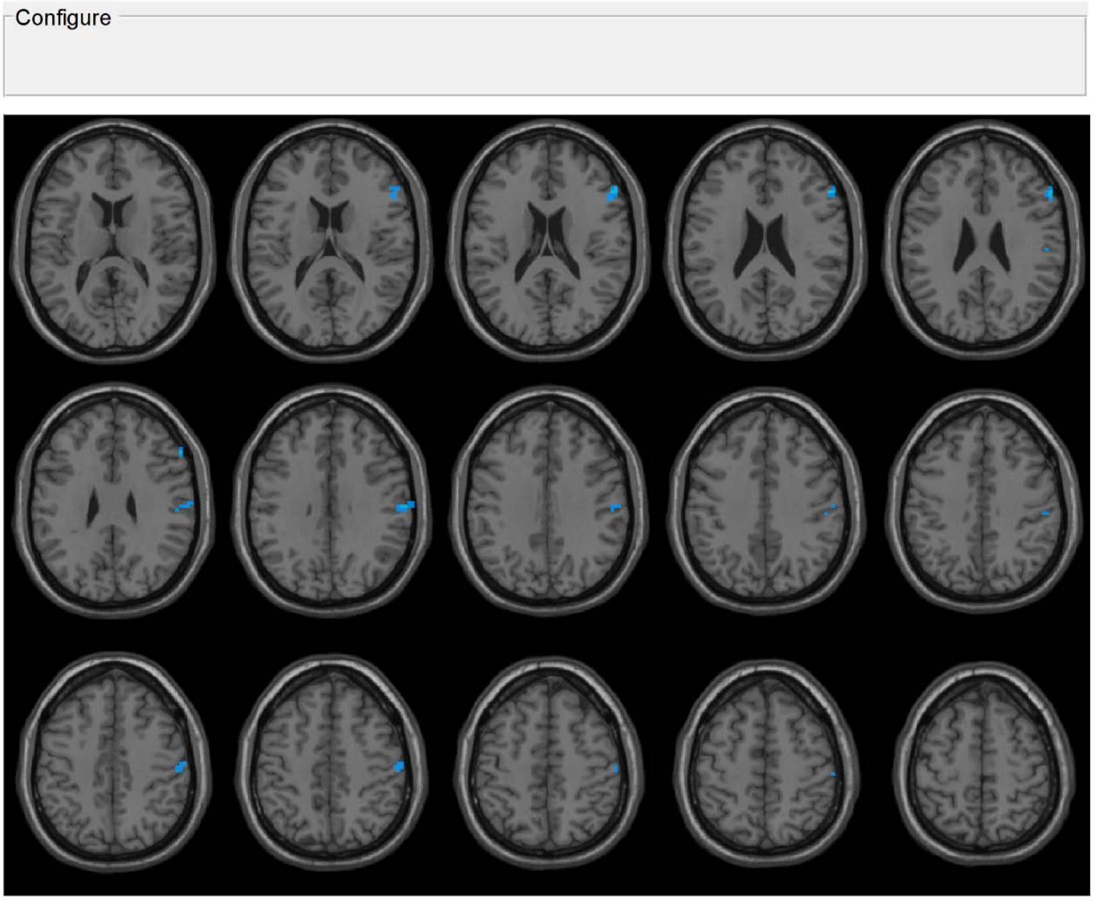

slow5 ReHo, full unedited image for Figure 1.

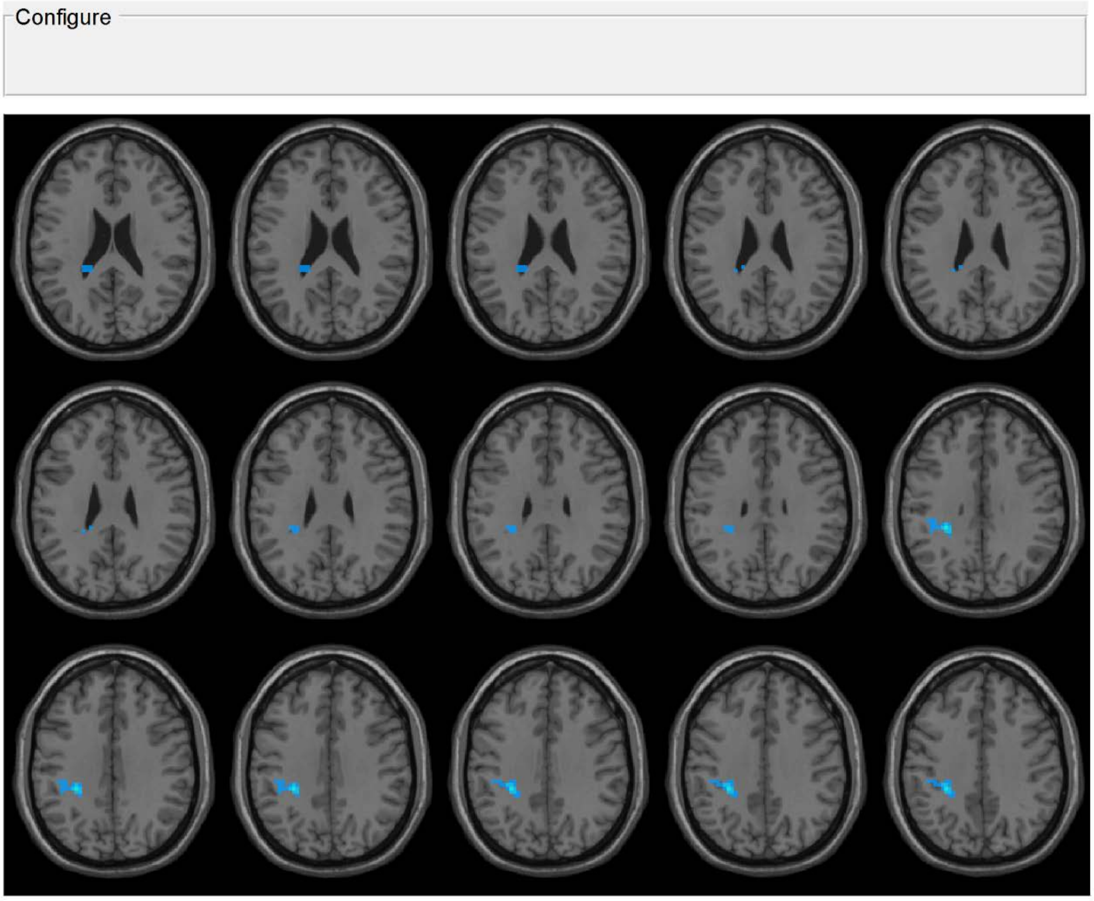

slow5 DC, full unedited image for Figure 1.

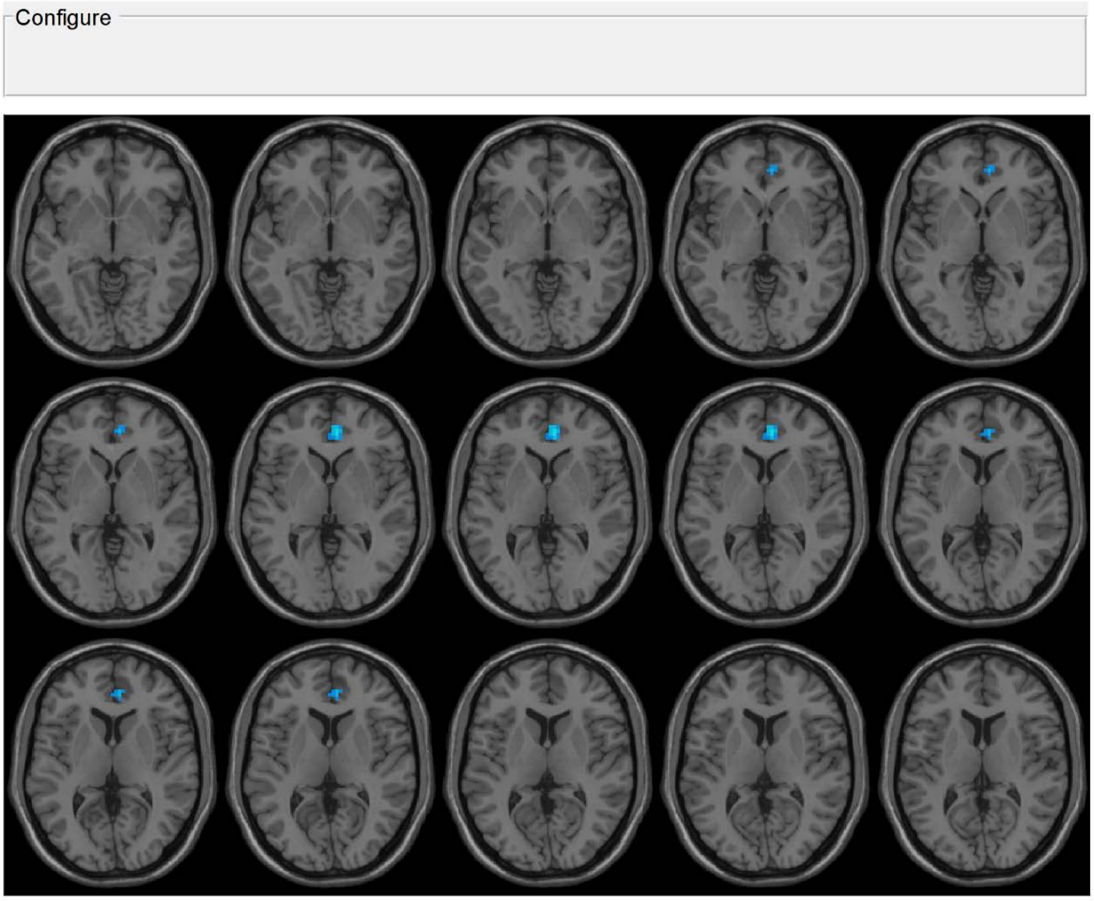

slow2 ALFF, full unedited image for Figure 1.

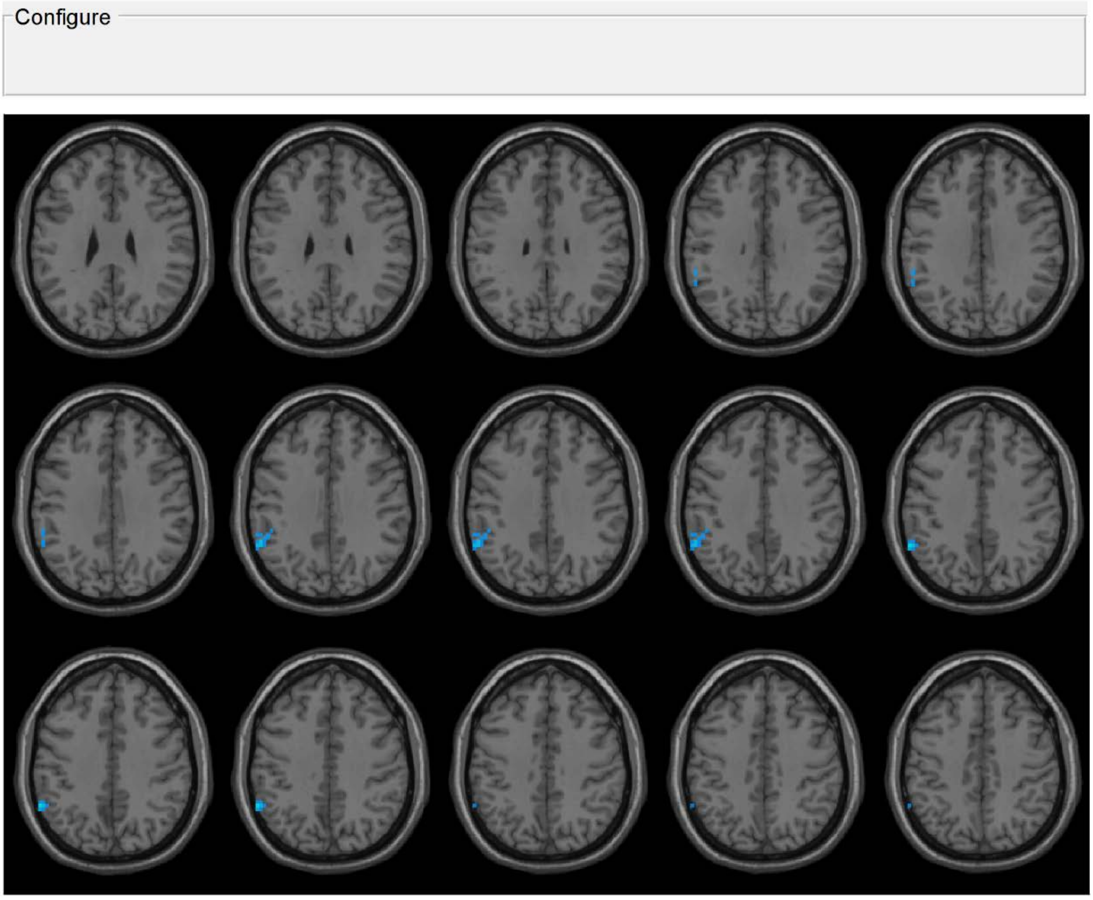

slow2 fALFF, full unedited image for Figure 1.

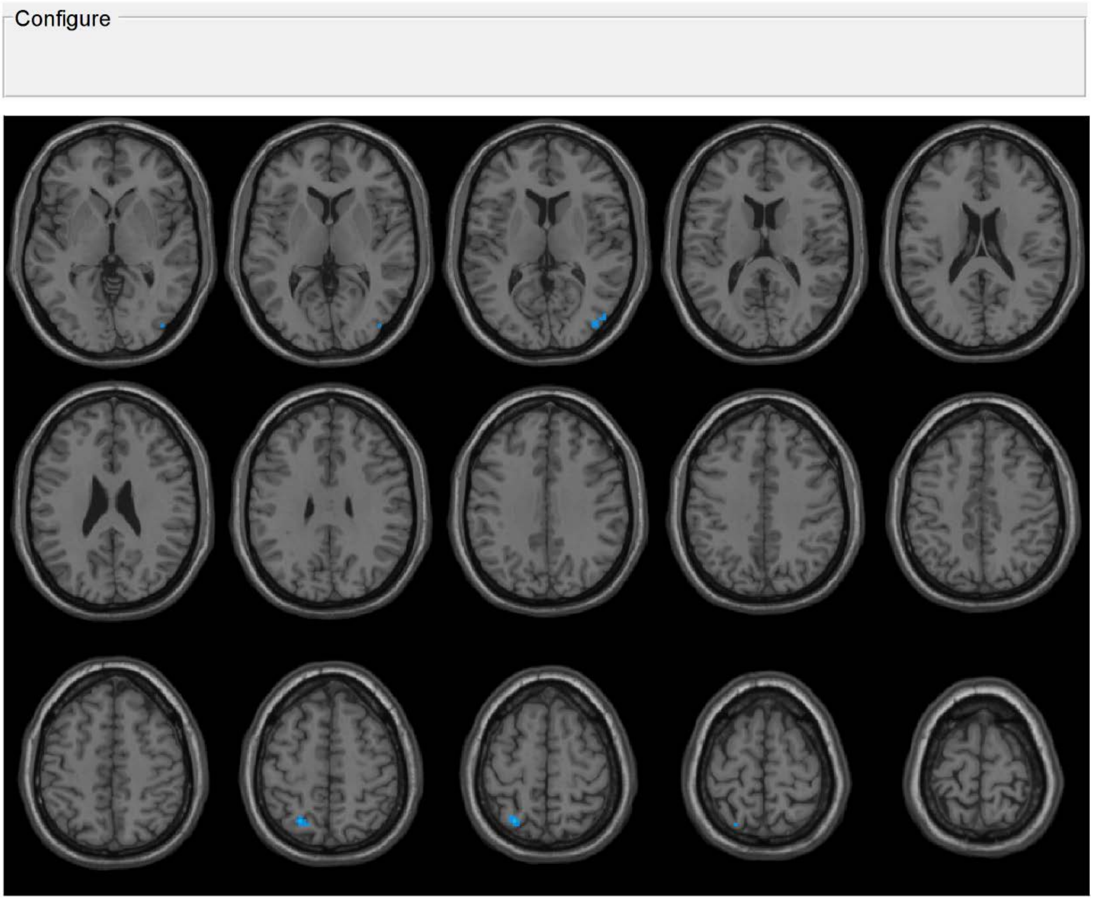

slow2 WaveletALFF, full unedited image for Figure 1.

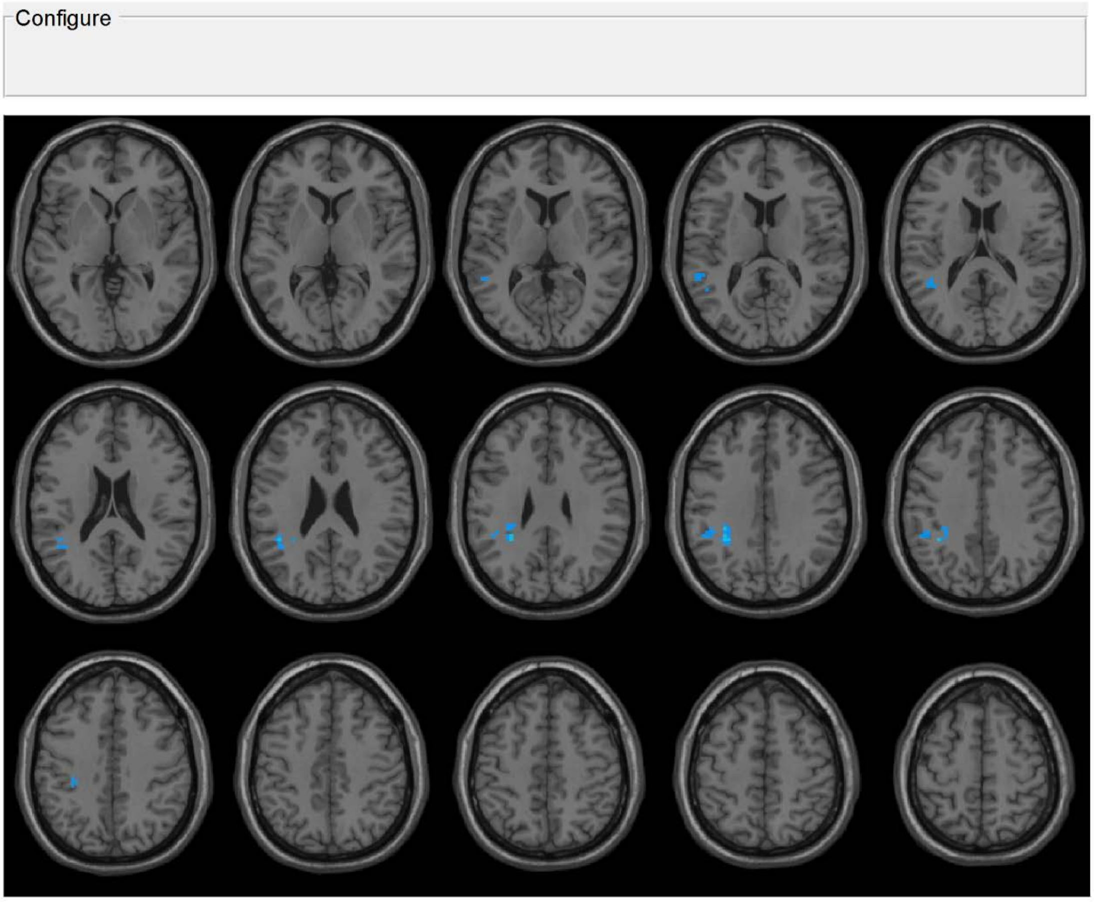

slow2 ReHo, full unedited image for Figure 1.

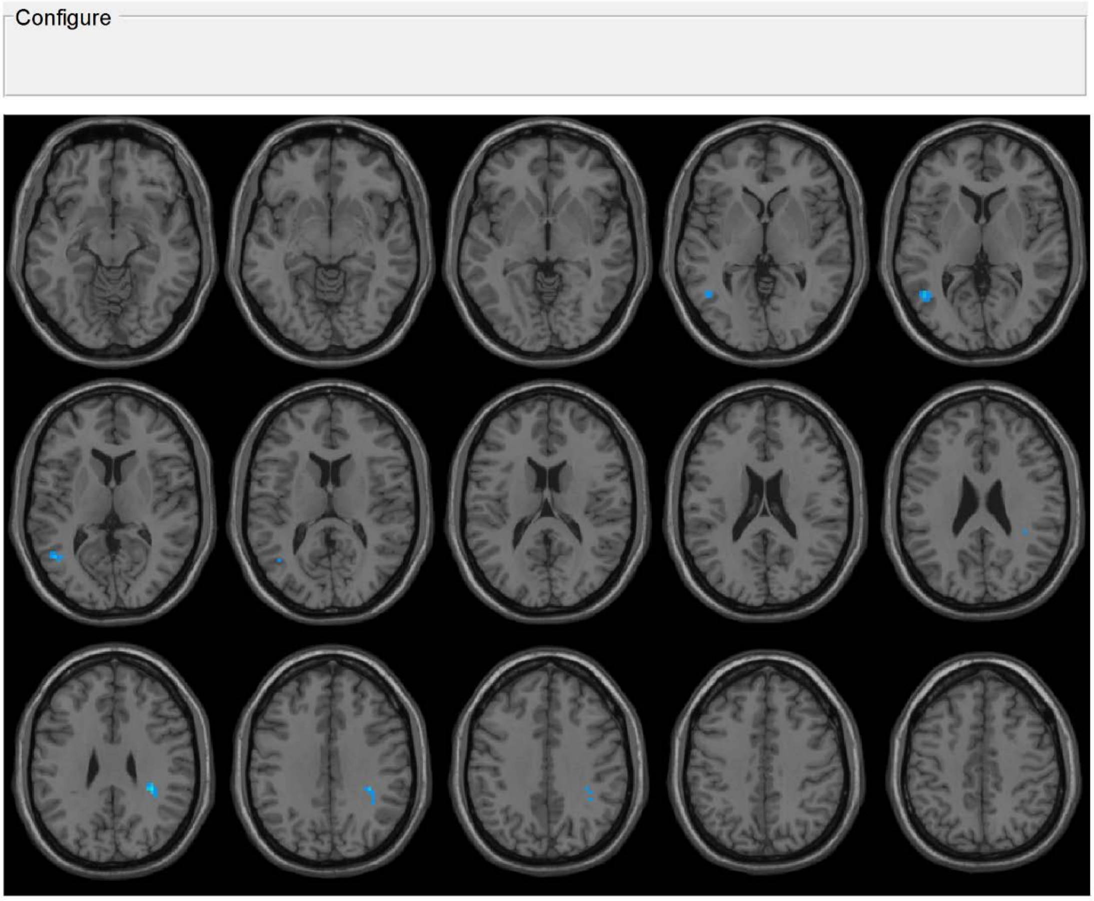

slow2 DC, full unedited image for Figure 1.

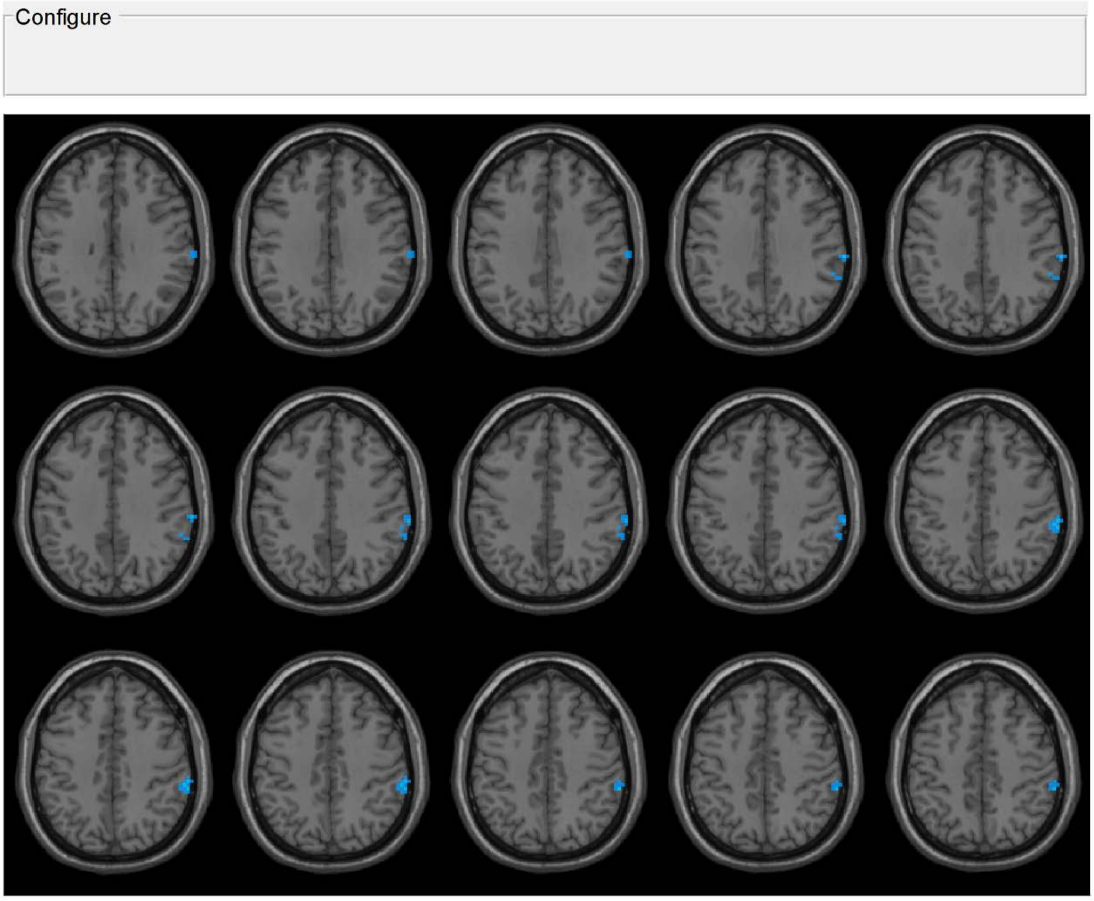

slow3 ALFF, full unedited image for Figure 2.

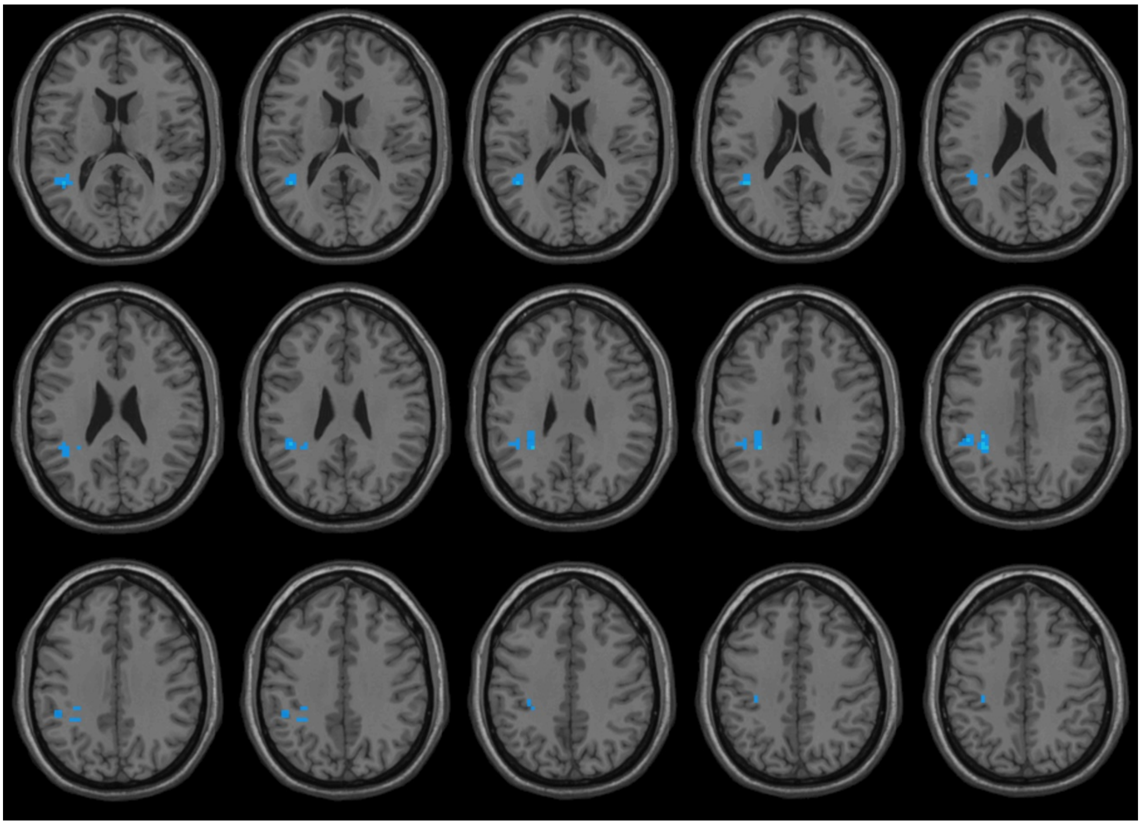

slow3 fALFF, full unedited image for Figure 2.

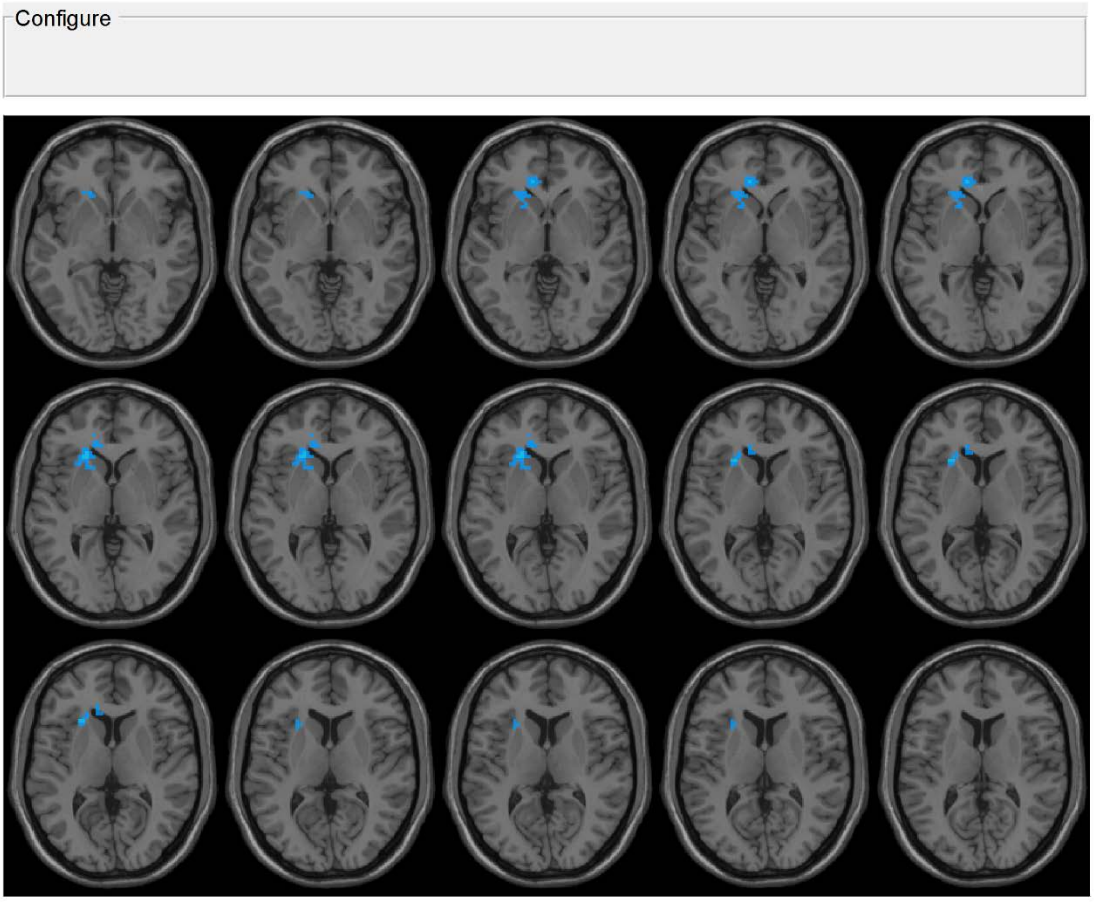

slow3 PerAF, full unedited image for Figure 2.

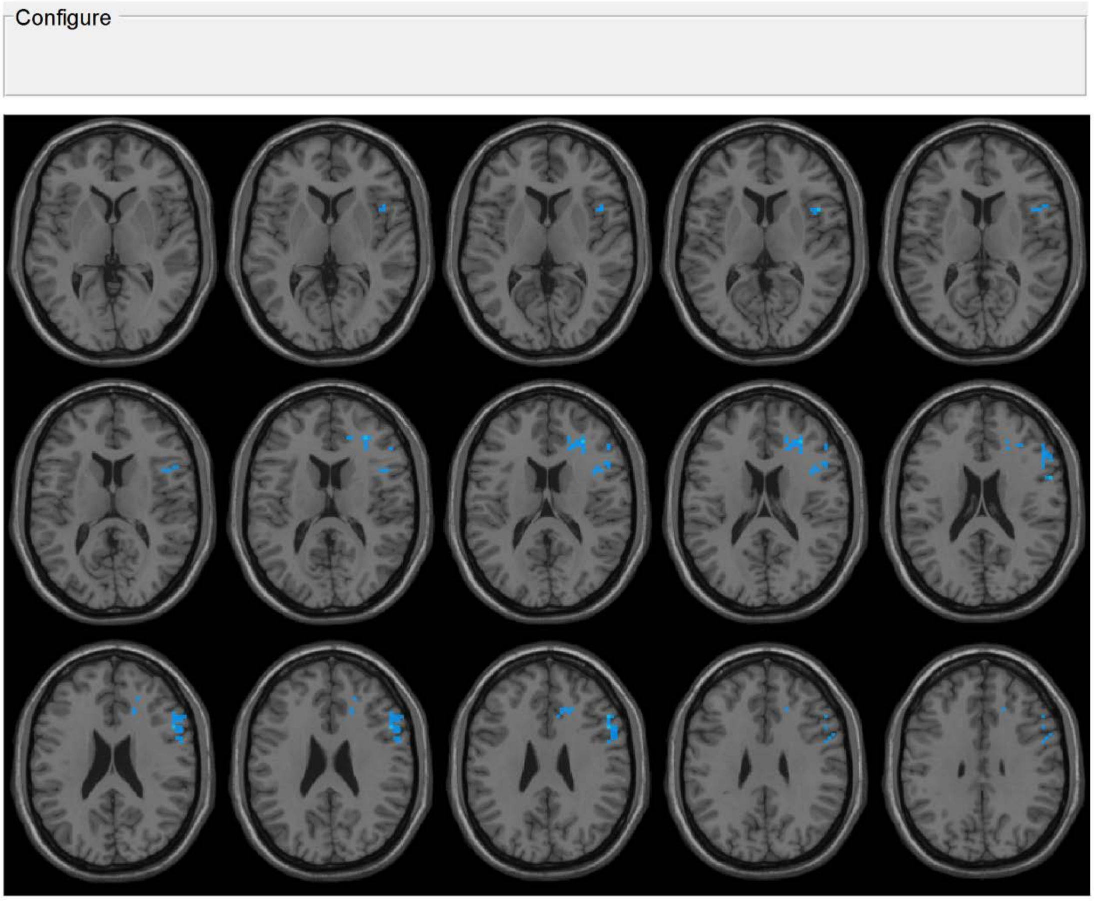

slow3 WaveletALFF, full unedited image for Figure 2.

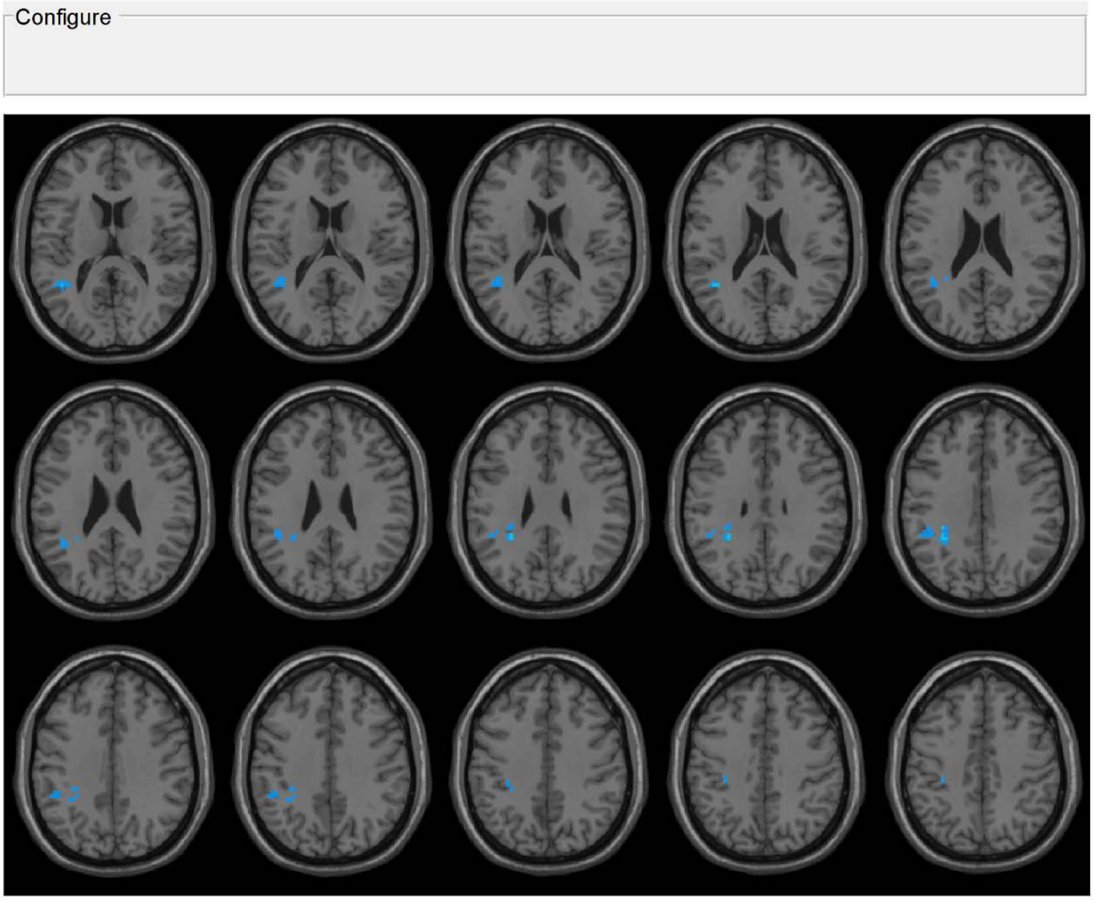

slow3 ReHo, full unedited image for Figure 2.

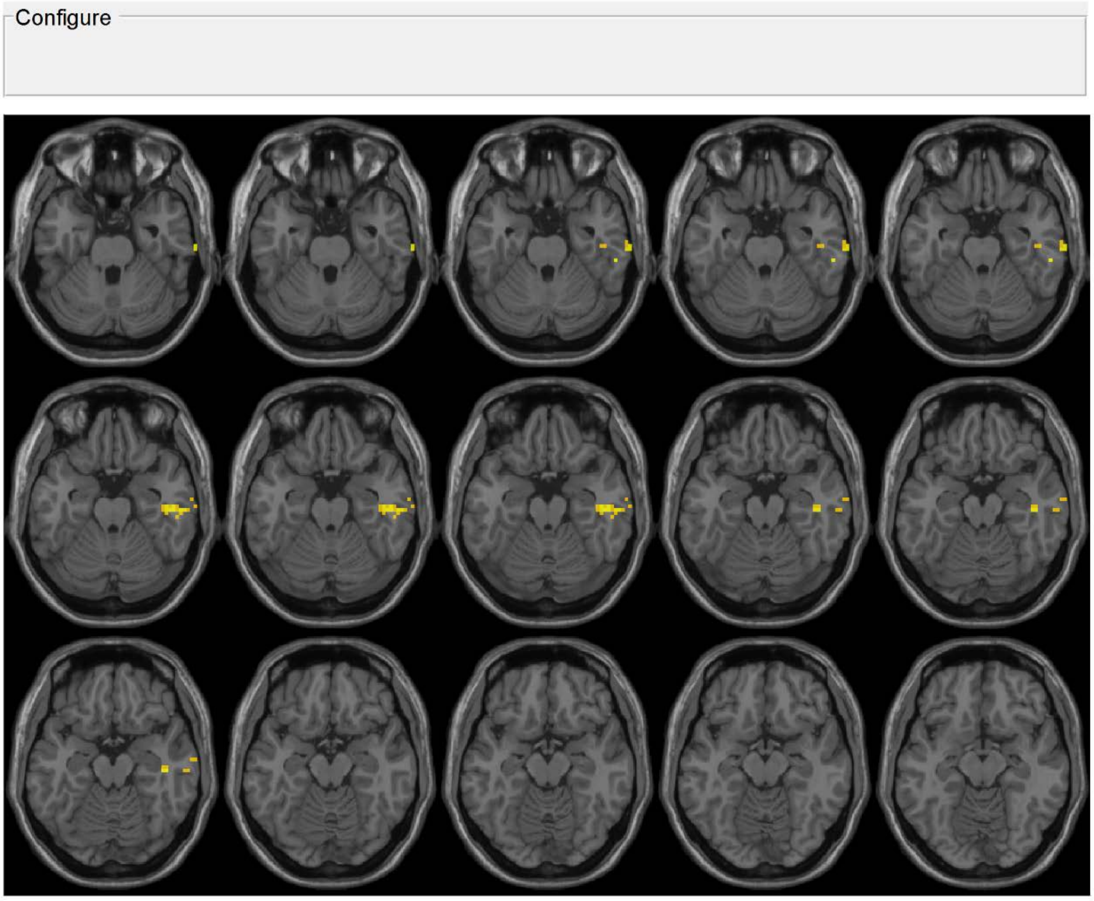

slow3 DC, full unedited image for Figure 2.

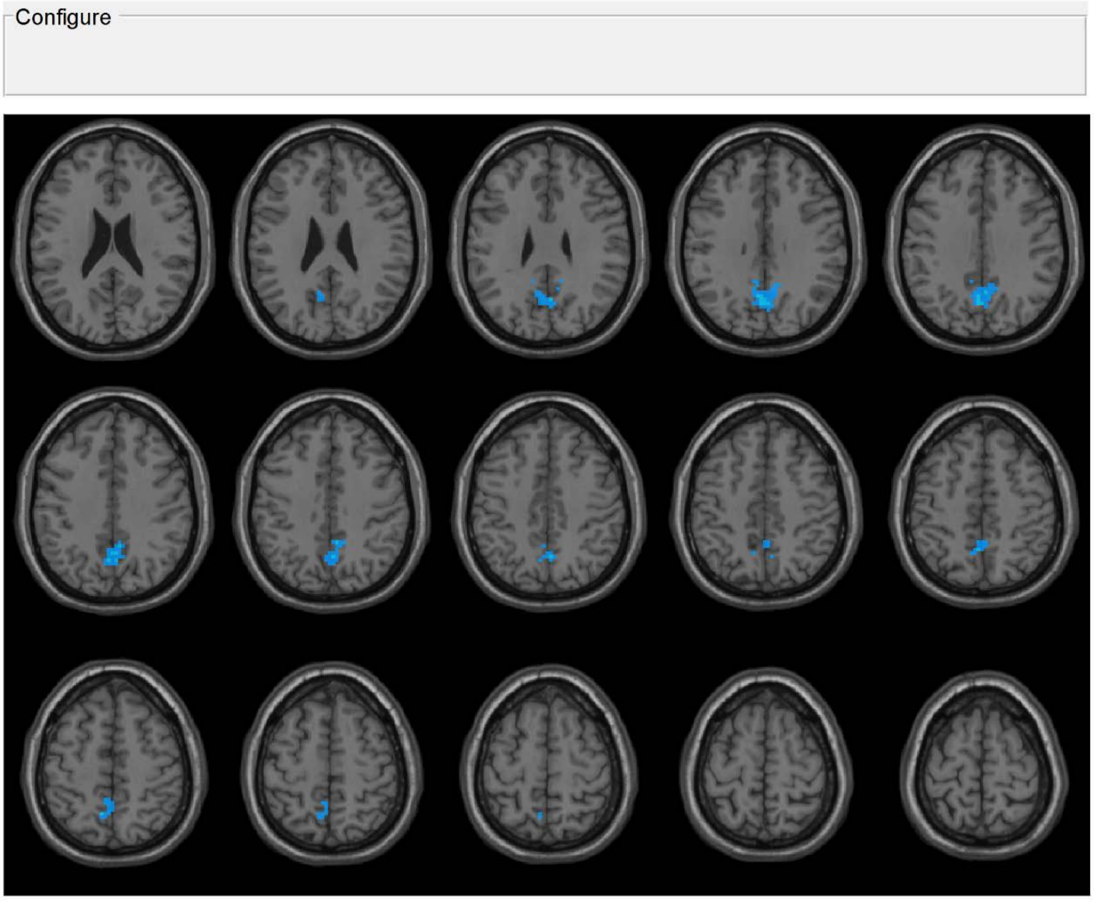

Supplementary file 2. T-Maps

The T-Maps resulting from fMRI analysis can be downloaded from the following URL: http://www.restfmri.net/MCI-SD/T-Map.zip
